# Author Correction: High-throughput prediction of protein conformational distributions with subsampled AlphaFold2

**DOI:** 10.1038/s41467-024-47504-0

**Published:** 2024-04-10

**Authors:** Gabriel Monteiro da Silva, Jennifer Y. Cui, David C. Dalgarno, George P. Lisi, Brenda M. Rubenstein

**Affiliations:** 1 Brown University Department of Molecular and Cell Biology and Biochemistry, Providence, RI USA; 2 Dalgarno Scientific LLC, Brookline, MA USA; 3 Brown University Department of Chemistry, Providence, RI USA

**Keywords:** Molecular conformation, Protein structure predictions, Computational biophysics

Correction to: *Nature Communications* 10.1038/s41467-024-46715-9, published online 27 March 2024

The original version of the Supplementary Information associated with this Article mistakenly omitted Supplementary Fig. 23. The HTML has been updated to include a corrected version of the Supplementary Information.

### Supplementary information

Updated Supplementary Information

