# Supplementary material for: High-throughput prediction of protein conformational distributions with subsampled AlphaFold2
Source: Nat Commun. 2024 Mar 27;15:2464. doi: 10.1038/s41467-024-46715-9 (PMC10973385; doi:10.1038/s41467-024-46715-9)
Supplement: Supplementary file 1 — Supplementary Information [file 41467_2024_46715_MOESM1_ESM.pdf]

Supplementary Materials for "High-Throughput Prediction of Protein Conformational Distributions with Subsampled AlphaFold2"

Gabriel Monteiro da Silva and Jennifer Y. Cui
Brown University Department of Molecular Biology, Cell Biology, and Biochemistry, Providence, RI, USA

David C. Dalgarno
Dalgarno Scientific LLC, Brookline, MA, USA

George P. Lisi and Brenda M. Rubenstein*
Brown University Department of Molecular Biology, Cell Biology, and Biochemistry
Brown University Department of Chemistry
Providence, RI, USA

April 1, 2024

AlphaFold 2 Subsampling and Abl1 Kinase Ensemble Predictions

The accuracy of our ensemble predictions was defined as their capacity to replicate the wild-type Abl1 kinase core conformations and their correct relative populations as validated by nuclear magnetic resonance experiments. Specifically, we sought a combination of parameters that led to an ensemble of predictions that met the following criteria: the ground state is the most frequent prediction within the ensemble, the transition from the ground to I2 state is captured within the ensemble, and the I2 state is present in the ensemble more frequently than transition states. Importantly, we opted to examine the relative populations of the ground and I2 states because of the large backbone rearrangement involved in the transition between these conformations, which is more likely to be reproduced by AF2 than the comparatively small dihedral flips in the ground to I1 transition. We optimized the accuracy achieved as a function of the following parameters: *max_seq*, *extra_seq*, number of seeds, and number of recycles (see Supplementary Table 1 for a complete list of tests and parameters). We evaluated the ensemble resulting from each parameter set by measuring the activation loop backbone RMSD relative to either the active kinase core (PDB 6XR6) [1] or the I2 kinase core (PDB 6XRG) [1] for each prediction. This decision is rooted in the fact that the activation loop is the structural element that changes the most (in terms of backbone motions) upon the transition from the ground to I2 state [1].

To encourage AF2 to generate a full ensemble of Abl1 conformations, we started by compiling an extensive MSA spanning over 600,000 sequences using the JackHMMR algorithm [2] on wild-type Abl1 kinase core (residues 229-515) sequences pulled from the UniRef90 [3], Small BFD [4], and MGnify [5] databases. To increase the statistical power of our results, we then ran 32 predictions with independent seeds for each test, and enabled dropouts during inference to sample from the uncertainty of the models. All other parameters were left in their default settings (3 recycles per prediction, 5 models per seed, a total of 160 predictions per run, 3 independent runs with unique seeds, 480 predictions per test).

In order to better quantify the effects of each parameter change, we binned each predicted structure into three classes based on the backbone RMSD of relevant structural elements (activation loop, phosphate-binding loop, and C helix) with respect to the backbone of these elements in the ground (active) state, as defined by the lowest-energy structure assignment in the NMR ensemble PDB 6XR6 [1]. Since the RMSD with respect to the ground state of the majority of predictions clustered within 3 Å, we classified predictions with RMSD values greater than 3.5 Å as “not in the ground state." See Supplementary Figure 1 for a depiction of the rationale behind this classification.

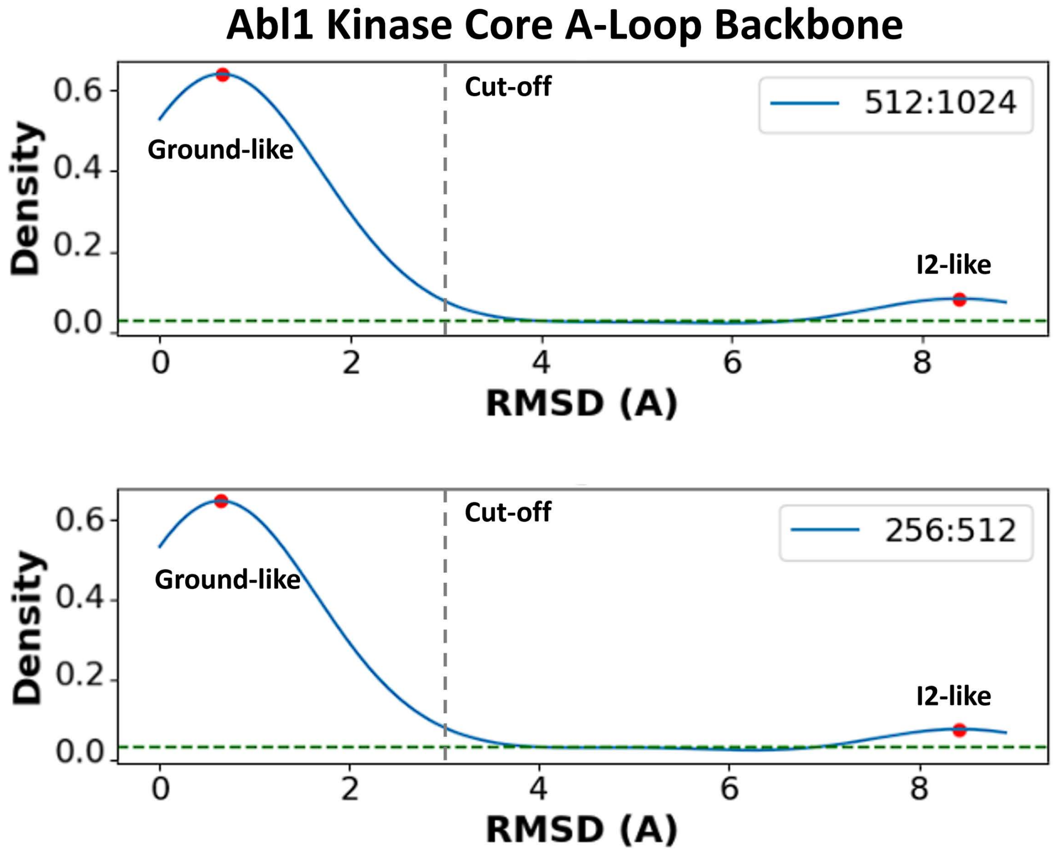

Supplementary Figure 1: of A-Loop (residues 379 to 395) backbone RMSD vs. the ground state reference (PDB 6XR6) for the predicted Abl1 kinase ensemble generated by AF2 with subsampling conditions (top) 512:1024 and (bottom) 256:512. Frequencies are calculated from a kernel density estimation with 480 samples per ensemble (96 independent seeds * 5 different models).

Through this binning, we observed that the 256:512 and 512:1024 values for *max_seq* and *extra_seq* led to predictions in which the ground state is populated 80% and 85% of the time, respectively. Of note, NMR results suggest that the relative state population of the Abl1 kinase core’s ground state in solution

is 88%, which is in surprisingly good agreement with our AF2 predictions [1]. In contrast with the effects observed from changing MSA composition and length, increasing the number of seeds beyond 128 did not lead to significant changes in the state distribution, suggesting a degree of determinism in the prediction results, presumably stemming from AF2 training biases and information encoded in the co-evolutionary signal (see Supplementary Figure 2).

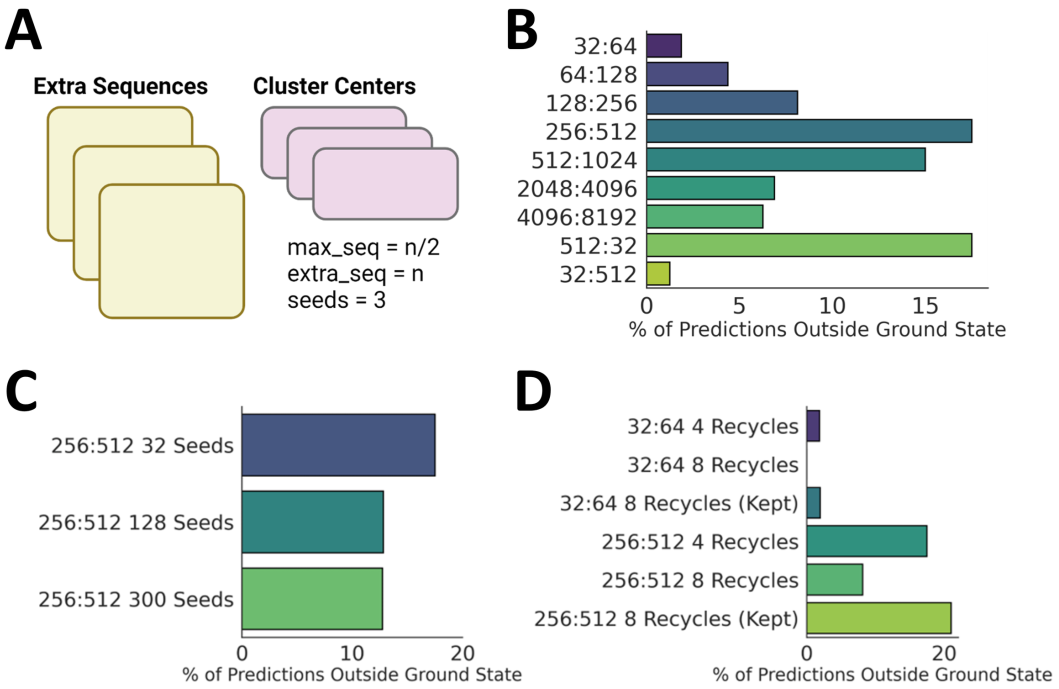

Supplementary Figure 2: Percent of Abl1 kinase domain conformations predicted to fall outside of the ground state using AF2 based on different MSA clustering parameters (number of sequences selected as cluster centers, number of sequences sampled from the clusters, number of seeds used in the prediction, and number of recycles). (A) Summary of the MSA subsampling and clustering algorithm implemented in AF2. (B) Percent of Abl1 kinase core conformations predicted to fall outside of the ground state using subsampled AF2 based upon the number of seeds used and the amount of recycling performed, and whether recycling intermediates are kept. (C) Impact of changing the number of seeds (each seed corresponds to an independent AF2 prediction); and (D) impact of changing the number of recycles and if structures from recycled iterations are included in the analysis or discarded. Each bar represents one data point (each data point is calculated from analyzing 160 measurements).

Interestingly, predictions with the *max_seq* and *extra_seq* parameters of 512 and 8, respectively, led to results that are similar to those of the 512:1024 test. Similarly, changing *max_seq* and *extra_seq* to 8:1024 led to results that closely resemble those from the 8:16 test. These results suggest that the *max_seq* parameter is the principal driver of alternative state predictions. This is unsurprising considering the different roles played by each parameter: the MSA of length defined by the *max_seq* argument and formed by the sequences randomly selected as cluster centers is passed to the expensive row/column attention Evoformer track, while the MSA of length *extra_seq* skips it. Due to the increased computational effort needed for featurization and attention, we expect AF2 to distill significantly more coevolutionary signal from the MSA of length *max_seq*, thus changes to *max_seq* will exert greater influence than changes than changes to *extra_seq*.

Finally, we also tested the hypothesis that changing the number of recycles (*n_recycles*) per seed could lead to changes in predicted state distributions by doubling the number of recycles. Interestingly, increasing the number of recycles significantly increases the population of the ground state, suggesting that the recycling stage plays a role in AF2's propensity to generate different conformations. Considering all of the above, we defined our target-specific parameters for all subsequent kinase predictions as follows: *max_seq*: 256, *extra_seq*: 512, *n_recycles*: 3, *n_models*: 5, *n_seeds*: 96. Considering its significant impact on the distribution of predictions, the optimization of the *max_seq* parameter is paramount for successfully obtaining conformational ensembles when running AF2. While 256 cluster centers (defined by *max_seq* = 256) works for Abl1, significantly smaller values are likely to be required for protein systems with less available sequence data.

Molecular Dynamics and WESTPA2 Simulations

Molecular dynamics simulations of wild-type Abl1 were conducted using the OpenMM software package [6] with the amber99sb-ildn force field [7] and the tip3p water model [8] at 300 K and 1 atm. The lowest energy Abl1 structure from the PDB 6XR6 [1] NMR ensemble was solvated within a dodecahedron box and charges were neutralized by replacing a number of solvent atoms with chloride and potassium ions. Following solvation, we minimized the energy of each system using a steepest-descent algorithm until the maximum force on any given atom was less than 1000 kJ/mol/min or until 50,000 minimization steps were conducted. We ran the simulations with a 1 fs time step during the equilibration phase and a 2 fs time step during the production phase. We equilibrated solvent atoms first for 1 ns in the NVT ensemble and then for 1 ns in the NPT ensemble with solute heavy atoms restrained using the LINCS algorithm with a spring constant of 1,000 kJ/mol/m^2 [9]. The production phase (in the NPT ensemble) followed the equilibration phase but without restraints.

We used the WESTPA2 [10] enhanced-sampling method to access the timescales necessary to simulate the inactivation pathway of Abl1. This was done via two WESTPA2 simulations (ground to I1 and I1 to I2). As progress coordinates for the ground to I1 transition, we defined the distance between the backbone oxygen of V299 and the center of mass of the carboxyl group of D381 as PC1; and the angle formed by the center of mass of the carboxyl group of D381, the backbone oxygen of K379, and the center of mass of the aromatic ring of F382 as PC2. For the I1 to I2 transition, we defined the distance between the backbone oxygen of L409 and the backbone oxygen of E377 as PC1; and the distance between backbone oxygen of L409 and the backbone oxygen of G4598 as PC2. Representative illustrations of the progress coordinates used in this protocol are in Supplementary Figure 3, and their distributions and start/end state definitions are described in Supplementary Figure 4. We ran WESTPA2 for 300 iterations for each leg of the transition, with the number of walkers per iteration varying from 64 to 512 due to the adaptive binning scheme, and 100 ps per iteration, totaling over 9 us of aggregate simulation time for each leg of the transition.

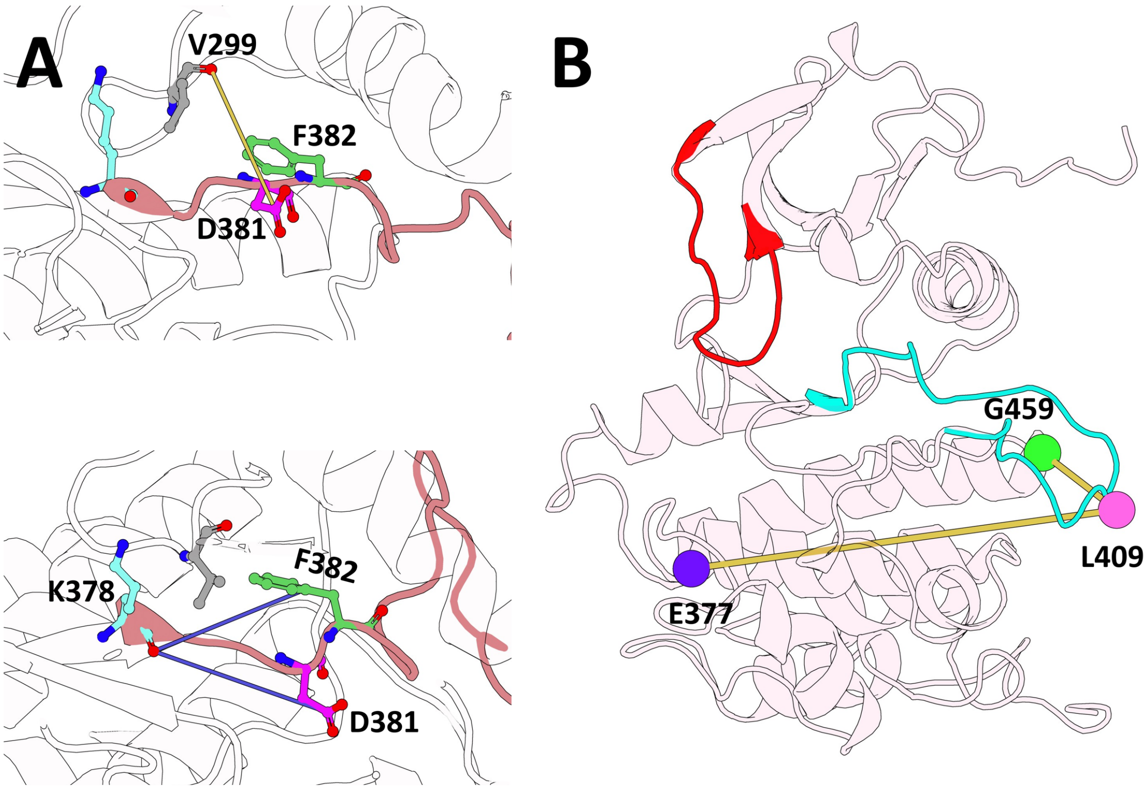

Supplementary Figure 3: Progress coordinates used in the WESTPA2 simulations of wild-type Abl1. (A) Progress coordinates used in sampling the transition from the ground to the I1 state. (B) Progress coordinates used in sampling the transition from the I1 to the I2 state.

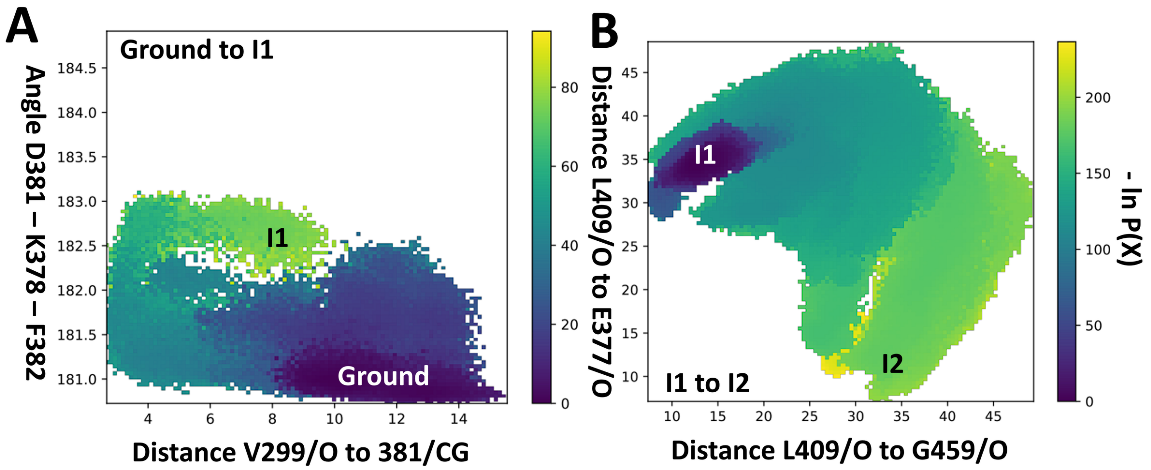

Supplementary Figure 4: Distribution of values for the progress coordinates used in either the transition from the (A) ground to the I1 state or (B) I1 to I2 state.

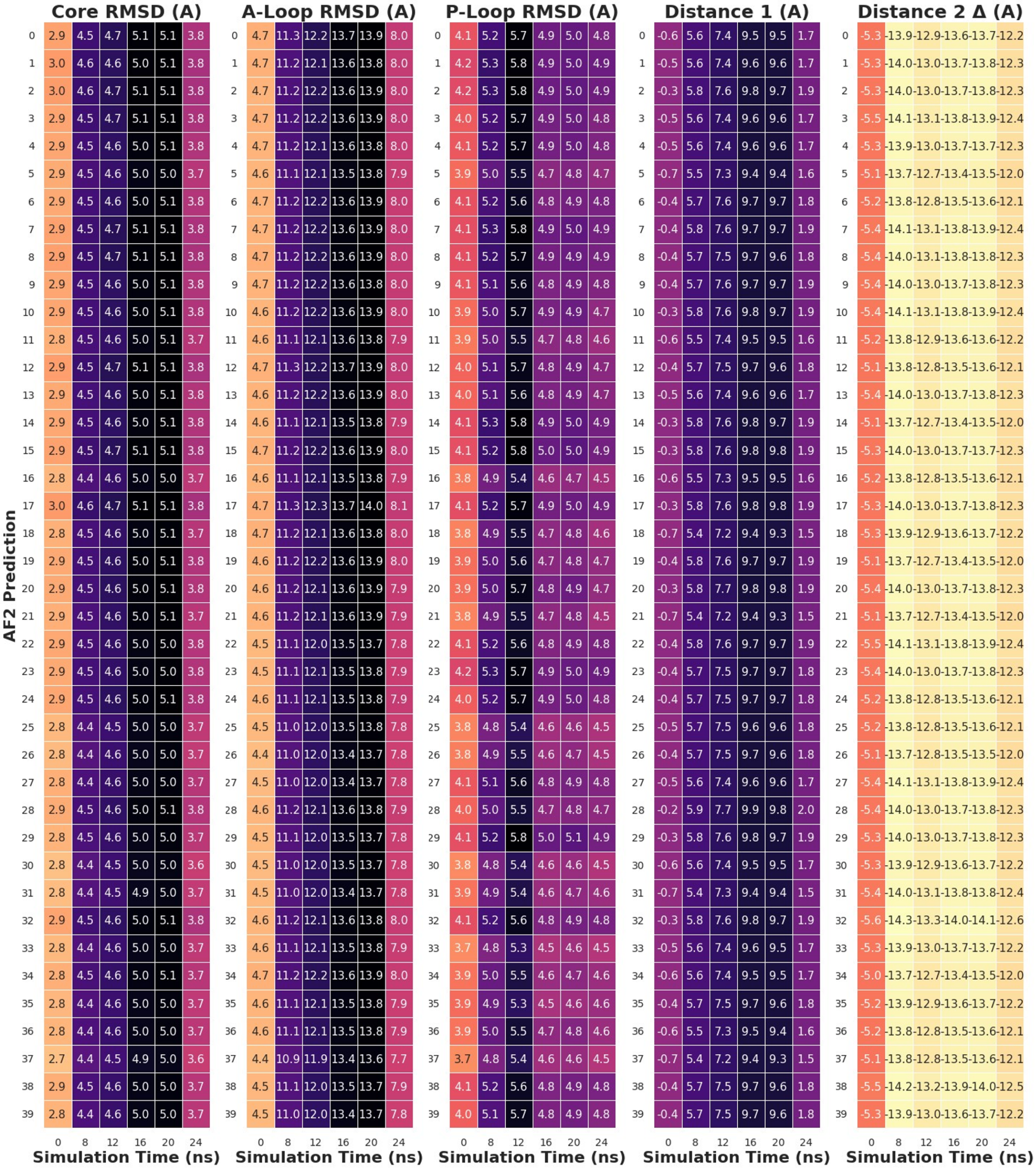

Supplementary Figure 5: Part one of four of the comparison between the values of five structural elements in the Abl1 kinase core known to change during the I1 to I2 transition as measured from the ensemble of 160 subsampled AF2 predictions and six frames extracted from a molecular dynamics simulation trajectory spanning the transition at different time points. Core, P-Loop, and A-Loop RMSDs are defined as the backbone RMSDs of each AF2 prediction's kinase core (residues 242 to 459), activation loop (residues 379 to 395), or phosphate-binding loop (residues 244 to 256) vs. the kinase core, phosphate-binding loop, or activation loop backbone of the MD snapshot selected at each time point. Distance deltas are defined as the difference in atom pair distances between each AF2 prediction and its respective MD snapshot. Distance 1 corresponds to the distance between the backbone oxygens of E377 and L409, and Distance 2 corresponds to the distance between the backbone oxygens of L409 and G457.

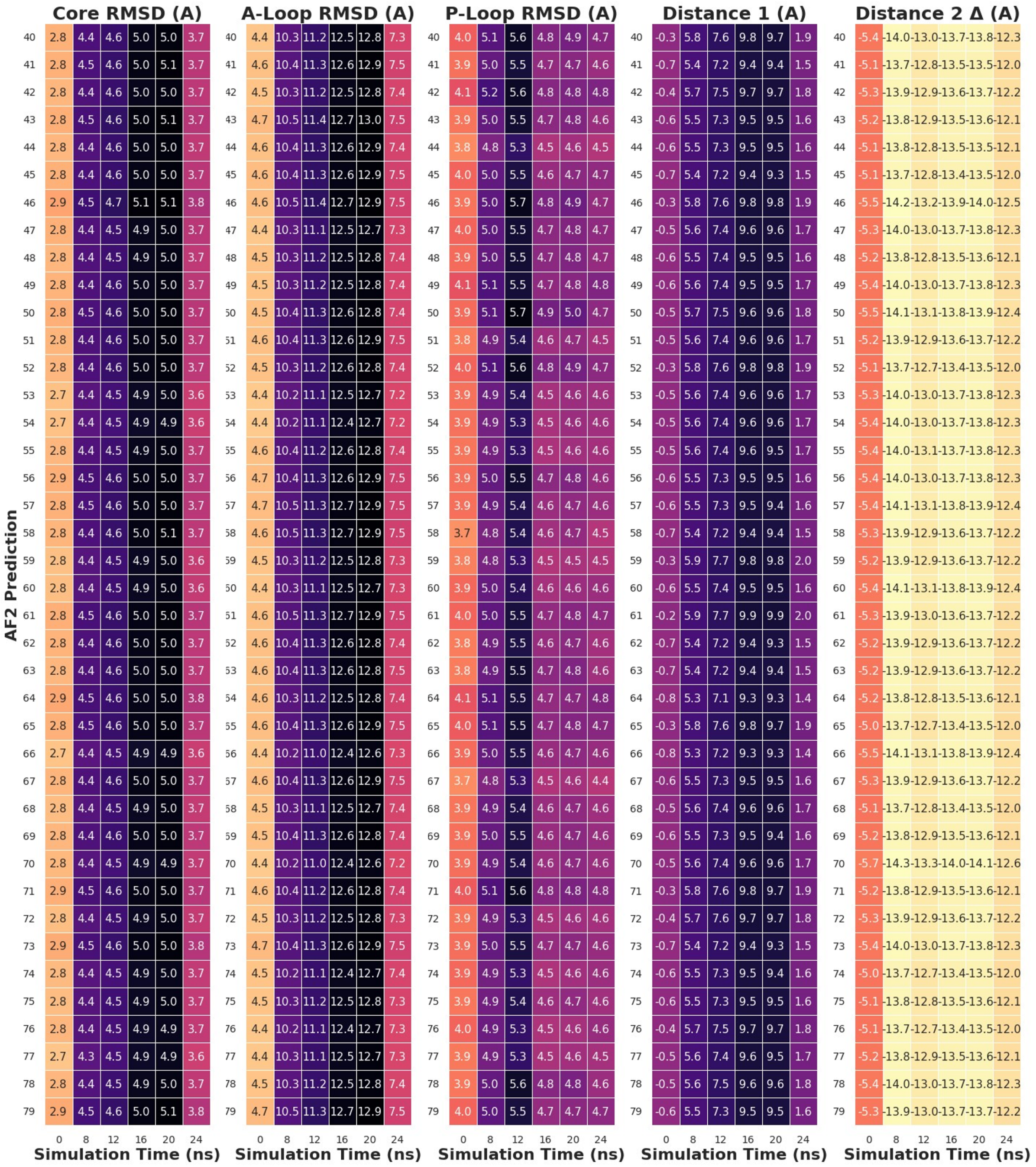

Supplementary Figure 6: Part two of four of the comparison between the values of five structural elements in the Abl1 kinase core known to change during the I1 to I2 transition as measured from the ensemble of 160 subsampled AF2 predictions and six frames extracted from a molecular dynamics simulation trajectory spanning the transition at different time points. Core, P-Loop, and A-Loop RMSDs are defined as the backbone RMSDs of each AF2 prediction's kinase core (residues 242 to 459), activation loop (residues 379 to 395), or phosphate-binding loop (residues 244 to 256) vs. the kinase core, phosphate-binding loop, or activation loop backbone of the MD snapshot selected at each time point. Distance deltas are defined as the difference in atom pair distances between each AF2 prediction and its respective MD snapshot. Distance 1 corresponds to the distance between the backbone oxygens of E377 and L409, and Distance 2 corresponds to the distance between the backbone oxygens of L409 and G457.

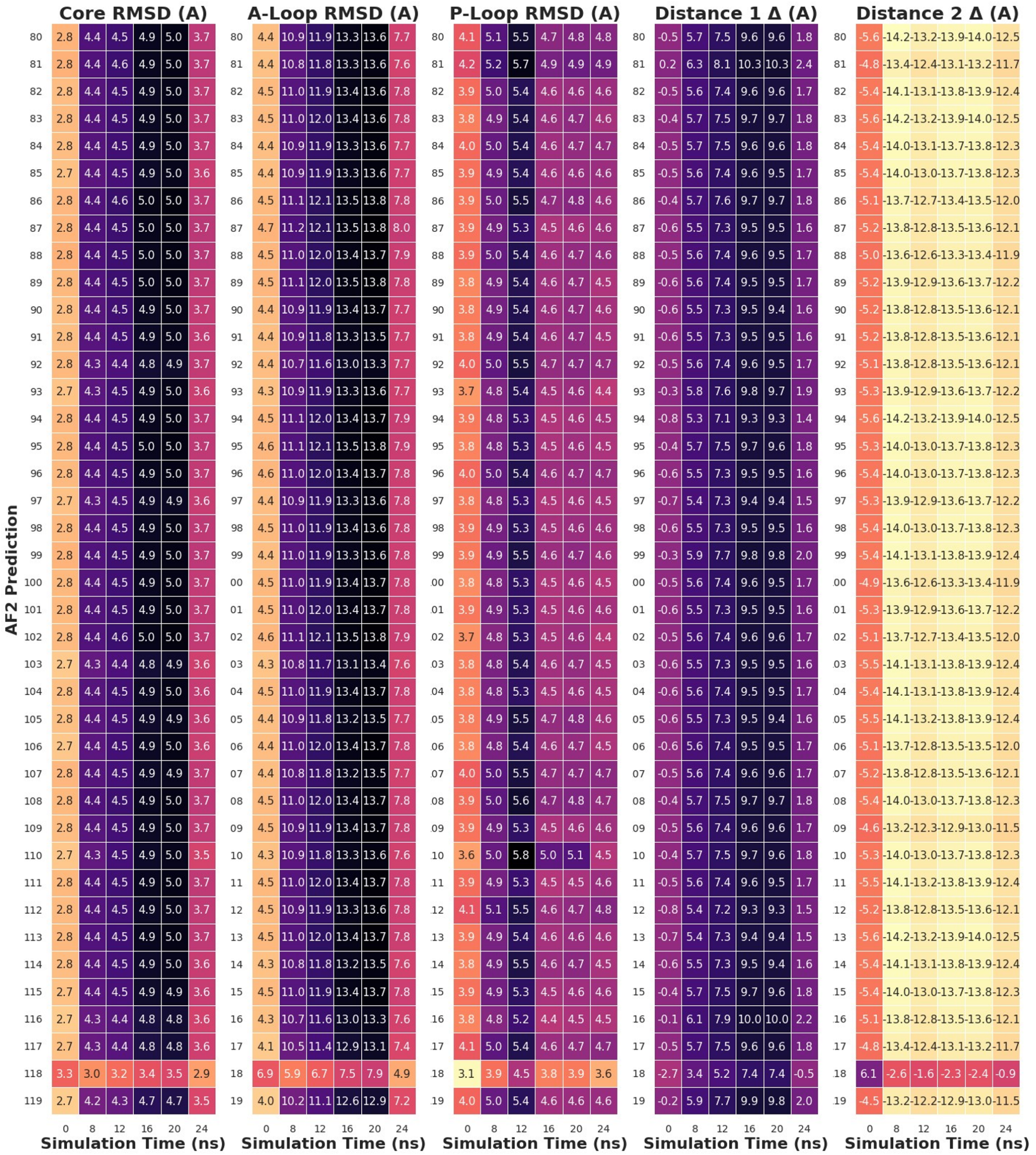

Supplementary Figure 7: Part three of four of the comparison between the values of five structural elements in the Abl1 kinase core known to change during the I1 to I2 transition as measured from the ensemble of 160 subsampled AF2 predictions and six frames extracted from a molecular dynamics simulation trajectory spanning the transition at different time points. Core, P-Loop, and A-Loop RMSDs are defined as the backbone RMSDs of each AF2 prediction's kinase core (residues 242 to 459), activation loop (residues 379 to 395), or phosphate-binding loop (residues 244 to 256) vs. the kinase core, phosphate-binding loop, or activation loop backbone of the MD snapshot selected at each time point. Distance deltas are defined as the difference in atom pair distances between each AF2 prediction and its respective MD snapshot. Distance 1 corresponds to the distance between the backbone oxygens of E377 and L409, and Distance 2 corresponds to the distance between the backbone oxygens of L409 and G457.

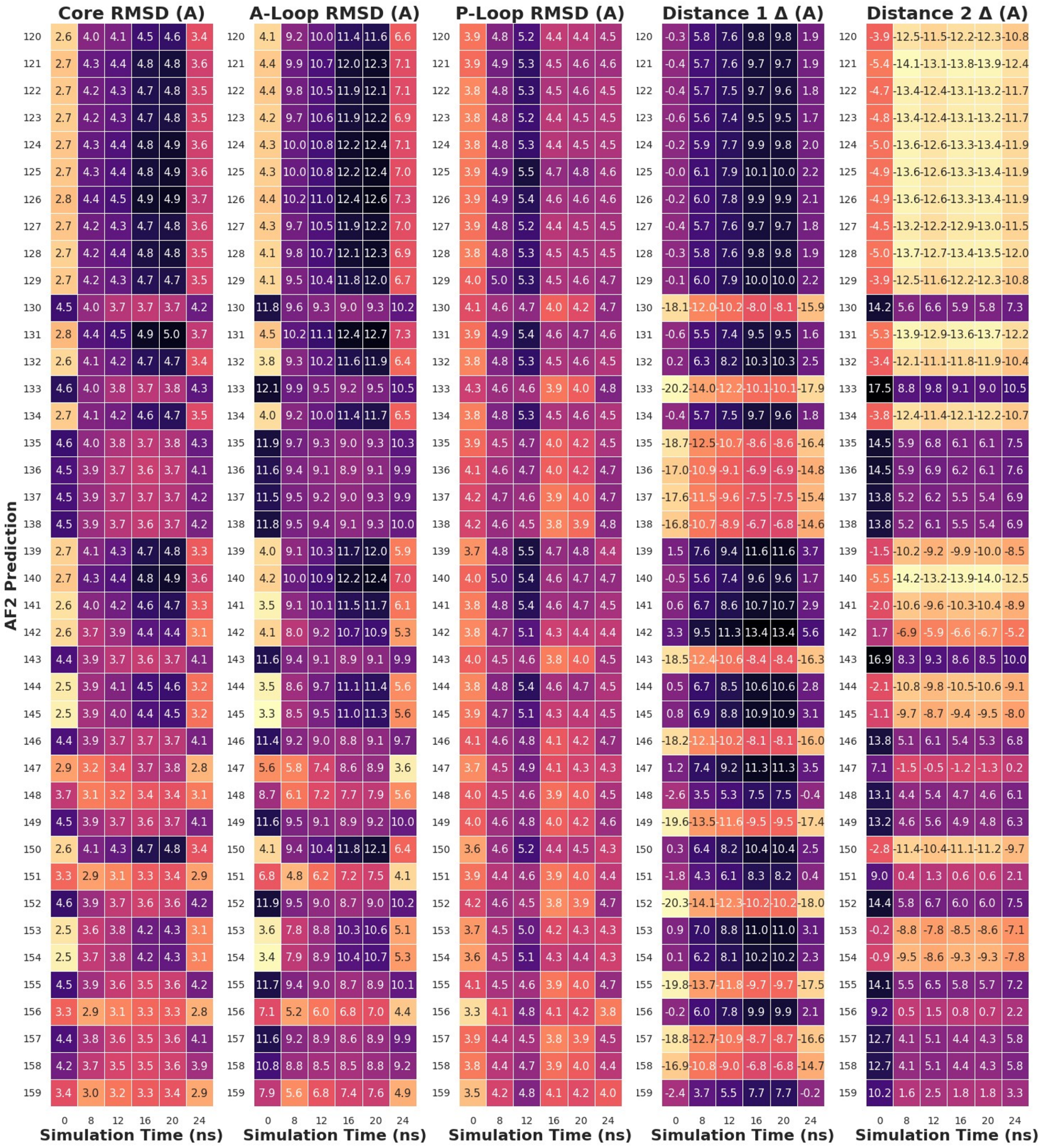

Supplementary Figure 8: Part four of four of the comparison between the values of five structural elements in the Abl1 kinase core known to change during the I1 to I2 transition as measured from the ensemble of 160 subsampled AF2 predictions and six frames extracted from a molecular dynamics simulation trajectory spanning the transition at different time points. Core, P-Loop, and A-Loop RMSDs are defined as the backbone RMSDs of each AF2 prediction's kinase core (residues 242 to 459), activation loop (residues 379 to 395), or phosphate-binding loop (residues 244 to 256) vs. the kinase core, phosphate-binding loop, or activation loop backbone of the MD snapshot selected at each time point. Distance deltas are defined as the difference in atom pair distances between each AF2 prediction and its respective MD snapshot. Distance 1 corresponds to the distance between the backbone oxygens of E377 and L409, and Distance 2 corresponds to the distance between the backbone oxygens of L409 and G457.

Abl1 Homolog Sequences Used to Generate Multiple Sequence Alignments

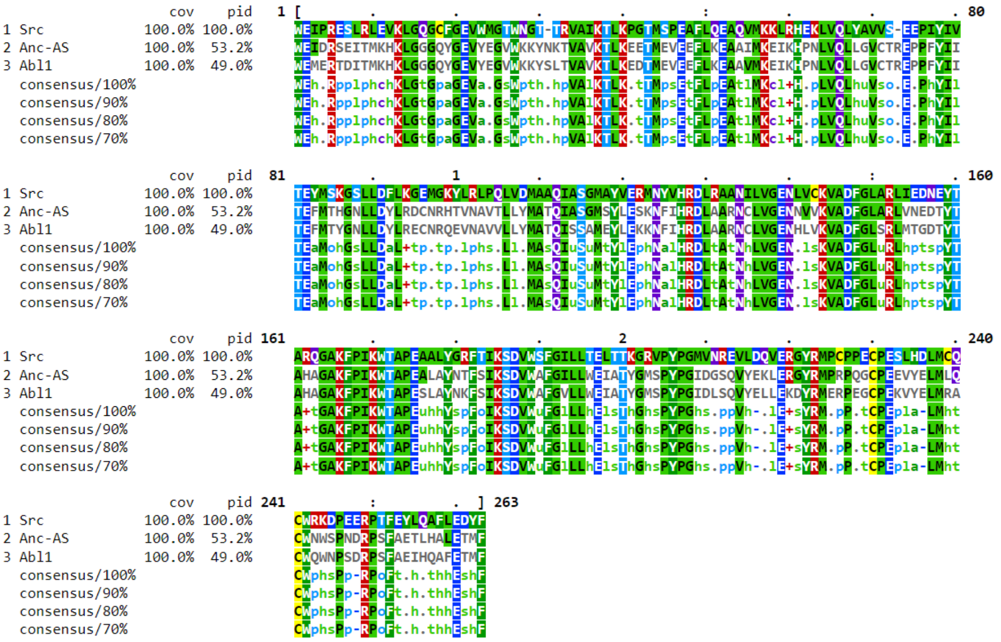

Supplementary Figure 9: Sequences of the Abl1, Src, and Anc-AS kinase cores used to generate MSAs as input for subsampled AlphaFold 2.

Optimization of AF2 Parameters for the Abl1 Protein

Supplementary Table 1: Optimized AF2 parameters for predicting Abl1 ensembles.

parameter_test	max_seq	extra_seq	n_recycles	n_models	n_seeds	%_notground
**t_max_extra_1**	32	64	3	5	32	2
**t_max_extra_2**	64	128	3	5	32	5
**t_max_extra_3**	128	256	3	5	32	9
**t_max_extra_4**	256	512	3	5	32	18
**t_max_extra_5**	512	1024	3	5	32	15
**t_max_extra_6**	2048	4096	3	5	32	7
**t_max_extra_7**	4098	8192	3	5	32	6
**t_max_extra_8**	512	32	3	5	32	18
**t_max_extra_9**	32	512	3	5	32	1
**t_nseeds_1**	256	512	3	5	128	12
**t_nseeds_2**	256	512	3	5	300	12
**t_nrecycles_1**	32	64	8	5	128	0
**t_nrecycles_2**	32	64	8 (kept)	5	128	2
**t_nrecycles_3**	256	512	8	5	128	8
**t_nrecycles_4**	256	512	8 (kept)	5	128	21

AF2 Predictions of the Relative State Populations of Abl1 Kinase Core Mutants

Supplementary Table 2: Abl1 kinase core mutants and their observed or expected effects on the relative populations of the active (Ground), inactive 1 (I1), or inactive 2 (I2) states.

	Ground	I1	I2
**Wild-Type**	88	6	6
**M290L**	55	10	35
**L301I**	25	10	65
**M290L + L301I**	8	10	82
**F382L**	90	0	10
**F382Y**	10	0	90
**F382V**	5	0	95
**I2M**	10	0	90
**E255V (I2M background)**	nr	nr	45
**T315I (I2M background)**	93	0	7
**E255V + T315i**	nr	nr	nr

Effects of Model Choice

In its current implementation, AF2 ships with five pre-trained models, which were trained for and applied in the CASP14 challenge [11, 12]. The differences between each model are slight, as they are all forked from the initial AF2 models. Namely, models (1, 2) were finely tuned with four templates, and models (3, 4, 5) did not use templates in their fine-tuning. Besides the use of templates, the models mostly diverge in the number of training times and the subsampling level used for training. Key differences among models are described in Supplementary Table 3.

Supplementary Table 3: Summary of differences among the five models shipped with AlphaFold2

Model	1	2	3	4	5
Init. From	N/A	1	N/A	3	3
N Templates	4	4	0	0	0
Max Nres	384	256	256	256	256
max_seq	512	512	512	512	512
extra_seq	5120	1024	5120	5120	1024
Training Samples	0.3*10e6	0.6*10e6	1.4*10e6	1.1*10e6	2.4*10e6
Training Time	20h	1d 13h	4d 1h	3d	5d 12h

To measure how each individual model fares at predicting the relative state populations of Abl1 and its activating and inactivating mutants, we divided Figure 6 into five plots, one for each model, and analyzed the accuracy (Supplementary Figure 10).

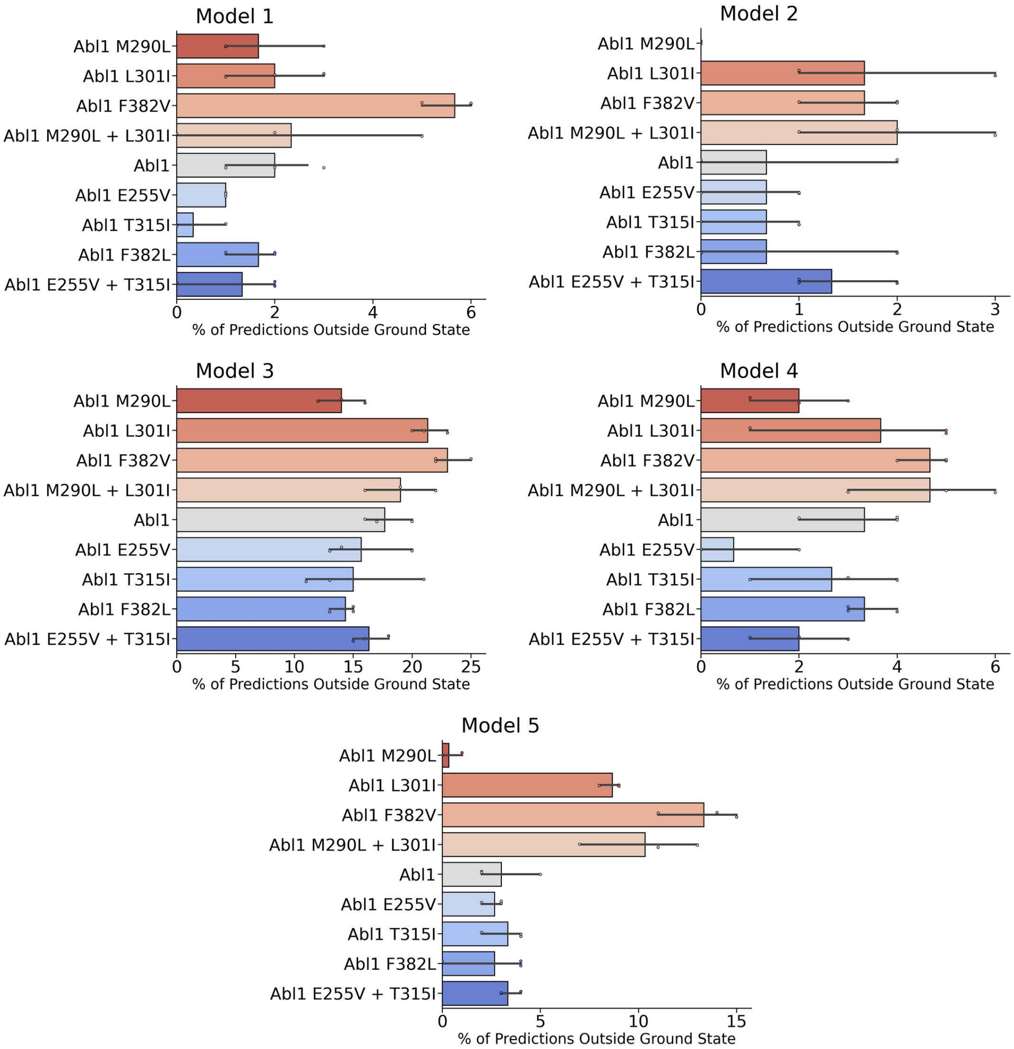

Supplementary Figure 10: Effects of model choice on predictions of the Abl1 activating and inactivating mutations. Each plot represents results from 96 independent seeds (32 seeds per replicate, 480 predictions in total across all five models), and error bars are calculated from the sets of triplicates. Predictions were considered outside of the ground state if their Activation Loop backbone RMSD vs. the ground state reference (PDB ID 6XR6) was above 3.5 Å. Data are presented as mean values +/- standard error of the mean.

Notably, models 3, 4, and 5 showed the best accuracy at predicting the effects of the Abl1 mutations, especially for the activating mutations. Models 3 and 5 showed the smallest variance, presumably due to the larger number of training samples used to generate them. Interestingly, all 5 models incorrectly predicted the M290L mutation as strongly inactivating, with models 5 and 2 leading to the most incorrect predictions. This unanimous inaccuracy suggests that the factors that lead to the Abl1 M290L mutants being incorrectly predicted potentially stem from other parts of the model not affected by the differences highlighted in Supplementary Table 3.

In summary, we observed significant differences in the accuracy of the predictions of relative state populations of Abl1 variants between the five models included in the current implementation of AF2. It is not in the scope of this study to explore which model is most appropriate for a given test case, but we anticipate that the observation that models that were refined in the absence of templates led to more

accurate predictions could be useful for further work seeking to answer this and related questions.

GMCSF Chemical Shift Perturbations

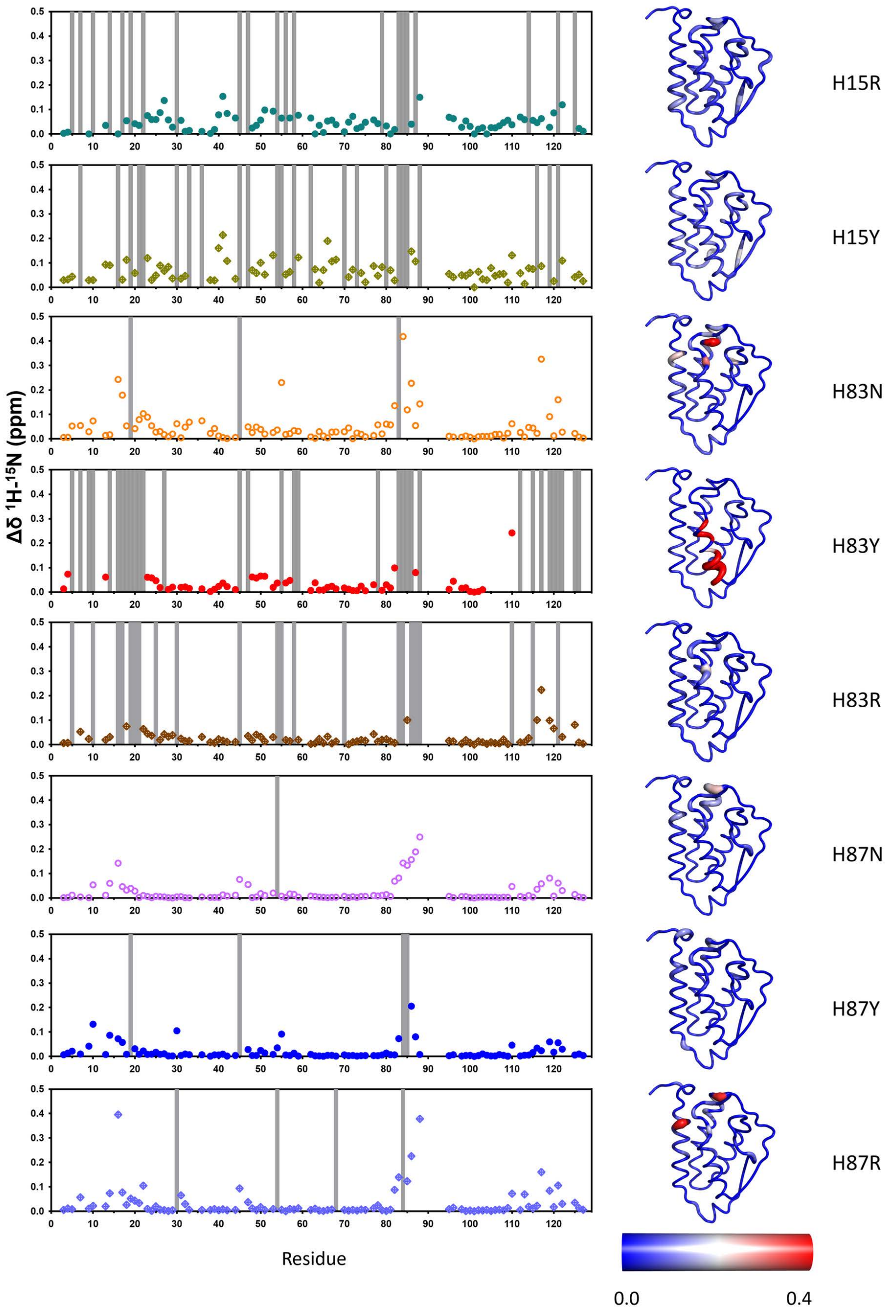

Supplementary Figure 11: ^{1}H-^{15}H Chemical shift perturbations for mutant GMCSF constructs relative to wild-type GMCSF peaks. Vertical bars indicate residues whose signal was lost due to chemical exchange broadening.

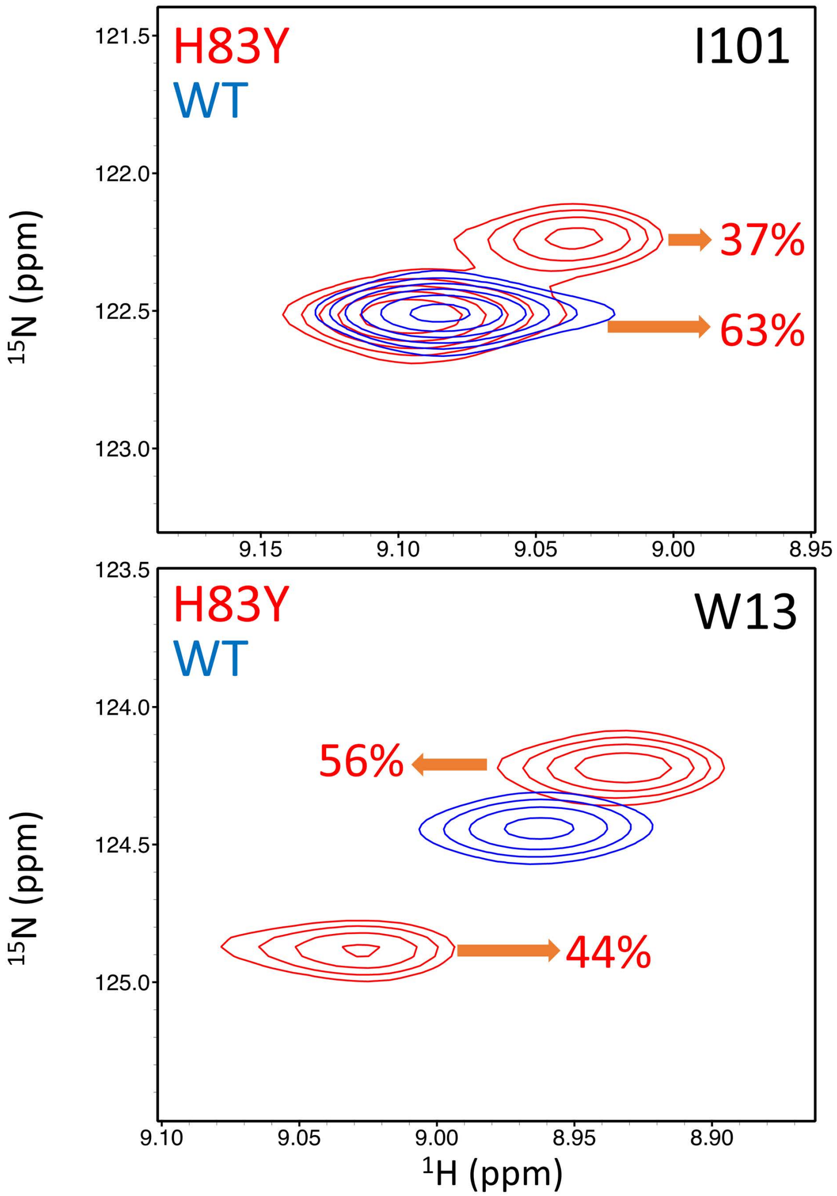

Supplementary Figure 12: Overlay of heteronuclear single quantum coherence measurements from WT GMCSF and the H83Y mutant showing residues experiencing slow exchange. The appearance of multiple resonances denotes a shift in the conformational exchange experienced at these residues in the mutant GMCSF. The relative populations of each conformer can be approximated by the resonance intensities (or volumes).

Optimization of AF2 Parameters for the GMCSF Protein

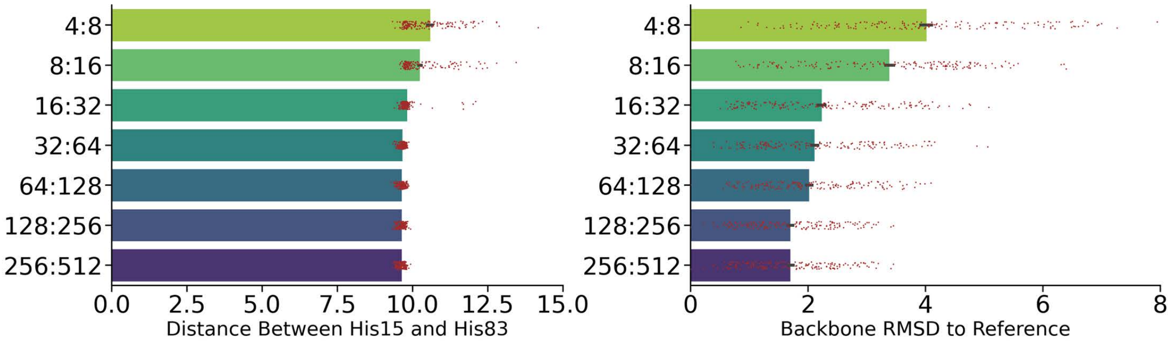

Supplementary Figure 13: Optimal AF2 subsampling parameters for GMCSF. (Left) Effects of modifying the *max_seqs* and *extra_seqs* values on the diversity of the distances between the H15 and H83 residues observed, which is a proxy for the opening of the heparin-binding site in GMCSF. (Right) Effects of modifying the *max_seqs* and *extra_seqs* values on the diversity of the root mean square deviation of atomic positions (RMSD) of the GMCSF backbone with respect to the ground state reference (the prediction closest to PDB 1CSG [13]). Data are presented as mean values +/- standard error of the mean.

GMCSF Conformational Ensemble Predictions

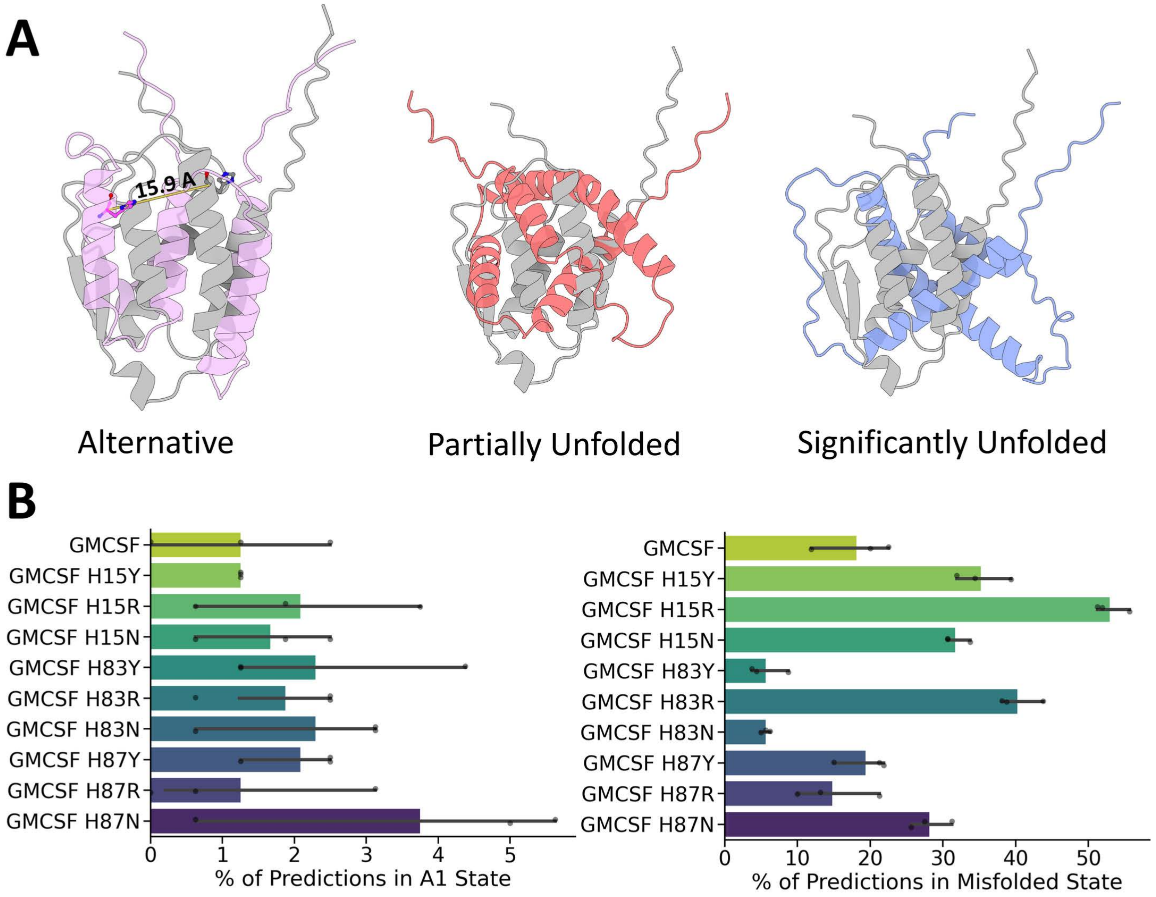

Supplementary Figure 14: Unusual GMCSF states predicted by subsampled AF2 and the respective populations of those states. (A) Structure of the most common alternative state predicted by AF2 (A1, in pink) aligned with and overlain on a ground state prediction (in grey). The distance between H83 in the reference and in conformation A1 is displayed as a measure of the difference between the conformations. Also shown are two misfolded/unfolded predictions aligned with and overlain on the ground state prediction (in grey). (B) AF2 predictions of the relative populations of the A1 conformation and the misfolded/unfolded structures. Conformations were classified as the A1 conformation based on the distance between the H15 and H83 residues (greater than 11 Å) and overall backbone RMSD vs. ground state reference (greater than 5 A but less than 10 Å), while they were classified as misfolded/unfolded based on overall backbone RMSD vs. the ground state reference (equal to or greater than 13 Å). Data are presented as mean values +/- standard error of the mean.

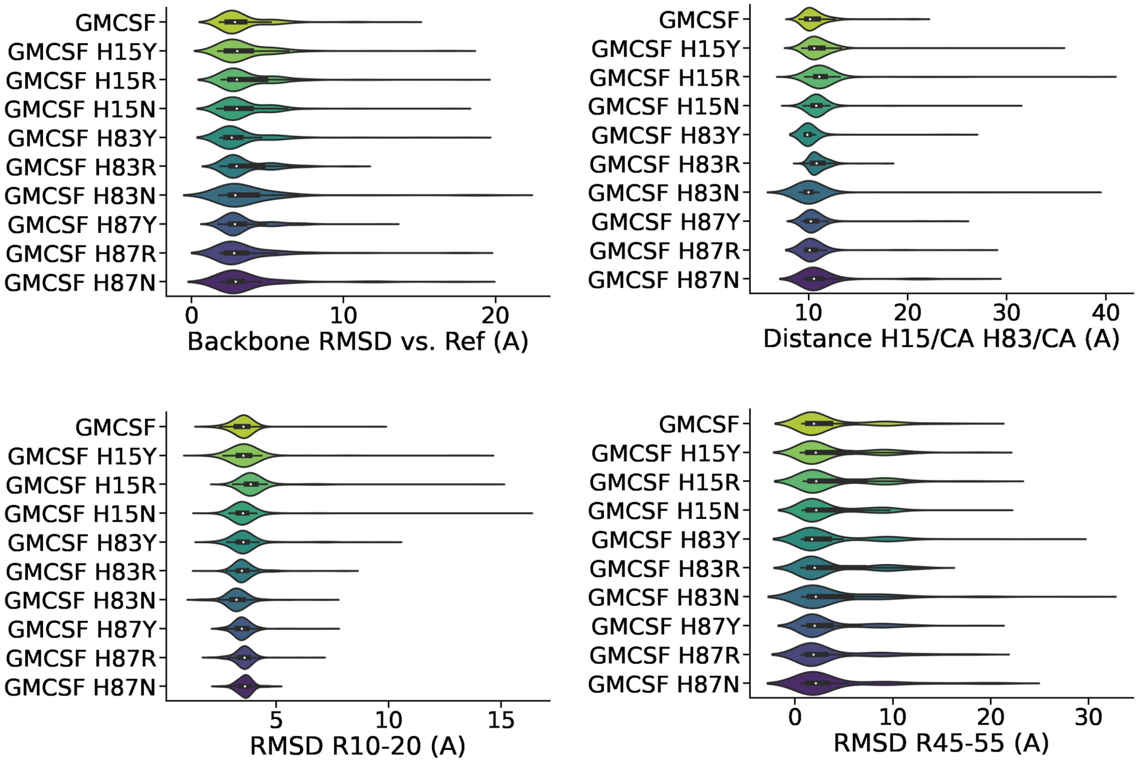

Supplementary Figure 15: AF2 predictions of the distributions of different GMCSF properties. Every RMSD measurement was taken with respect to the ground state reference (the prediction closest to PDB 1CSG).

Supplementary Appendix: Additional Test Cases

In order to measure the potential of our subsampled AF2 approach as a general tool for predicting the alternative conformations of proteins and their relative populations, we curated a test set of eight proteins with significantly different functions, lengths, conformational landscapes, and evolutionary histories. The composition of this test set is summarized below in Supplementary Table 4.

Supplementary Table 4: Additional test set composition.

**Example**	**Measurement**	**Ref. 1**	**Ref. 2**	**MSA Depth**
**Calmodulin**	Backbone	Bent linker (4BW7b)	Extended linker (4BW8b)	77210
**Fyn SH3**	C-Term. BB	Ordered C-Term. (Pred.)	Disordered C-Term. (Pred.)	50521
**AlkB**	Binding site BB	Closed (3I49a)	Open (Pred.)	14236
**CCR5**	Backbone	Inactive (5UIWa)	Active (7F1Qr)	62583
**LmrP**	Backbone	Outward-Facing (6T1Za)	Inward-Facing (Pred.)	4724
**LAT1**	Backbone	Inward-Facing (6IRSb)	Outward-Facing (7DSQb)	53314
**Aurora A**	A-Loop BB	Ground/Active (6XR6a)	I2/Inactive (6XRGa)	820754
**C. Anhyd. IV**	Backbone	Ground (3FE4a)	Ground (Pred.)	40033

Importantly, all the proteins in the test set with the exception of Carbonic Anhydrase (which is included as a negative control) are known to occupy distinct conformational states. Three proteins in the test set have been previously studied in other AlphaFold subsampling studies (LmrP, LAT1, and CCR5), with mixed results [14]. Below, we describe our prediction results in detail for each protein in the test set.

CCR5

The C-C Chemokine Receptor Type 5 (CCR5) is an immune system protein expressed on the surface of white blood cells [15]. Previous studies seeking to predict different conformations of CCR5 using subsampled AlphaFold were not successful in predicting significantly different alternative conformations of CCR5, such as the active conformation shown in PDB 7F1Qr [14, 16]. This lack of resolution in subsampled AF was attributed to biases introduced by the training set composition, as the alternative conformation was published in the PDB in 2021 and thus was not included in AlphaFold's training set [16].

To test if our subsampled AF2 approach fared any better than previous attempts at predicting alternative conformations of CCR5, we made a series of predictions of CCR5 with different subsampling conditions ranging from 4:8 to 1024:2048 (max_seq:extra_seq), with a total of 480 individual predictions for each subsampling condition (96 seeds times five models). The results of these predictions are summarized in Supplementary Figure 16.

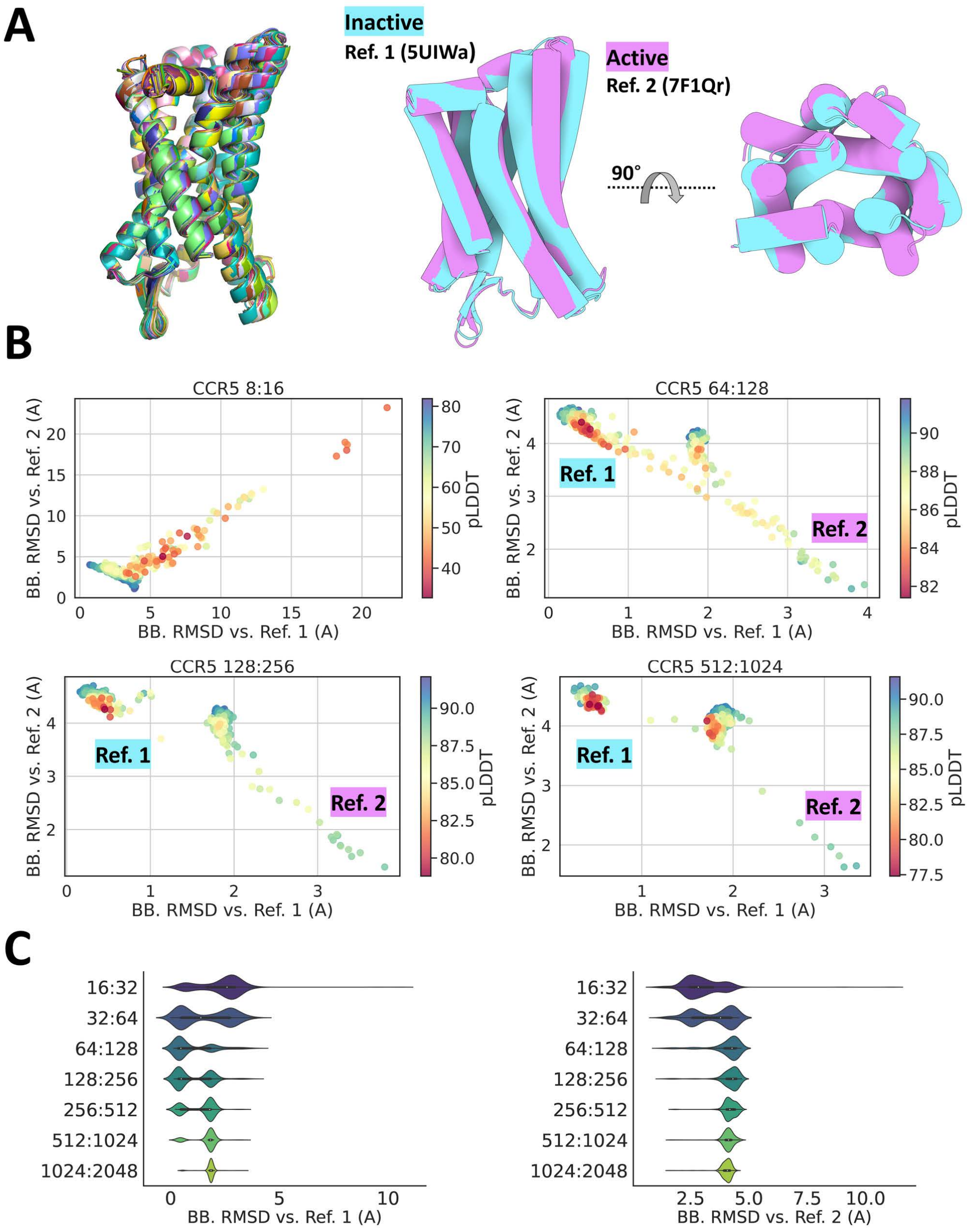

Supplementary Figure 16: Predictions for the CCR5 system using the subsampled AF2 methodology described in this study. (A) (left) Structural models obtained from the prediction method, aligned to the top-ranked prediction by pLDDT (AF2's confidence metric) and overlaid on top of each other in different colors; (middle) Rendering of the conformational references used to summarize the prediction results in the backbone RMSD vs. references scatterplots, each structure is colored according to its accompanying label; and (right) Alternate view of the structural references. (B) Bidimensional projection of four sample prediction results, comparing the similarity of each prediction to either Ref. 1 (inactive, PDB ID 5UIWa) or Ref. 2 (active, PDB ID 7F1Qr) by a backbone RMSD metric. Predictions are colored by average pLDDT, which is a metric of AlphaFold2's confidence in the resulting model. (C) Distribution of backbone RMSD values vs. each reference for each subsampling condition tested (conditions 4:8 through 16:32 omitted from the plot to avoid distortion of the X axis).

Surprisingly and in contrast with previous methods, our approach leads to predictions of CCR5 both in the ground and alternative state with most subsampling conditions, with good coverage of in-between conformations along the putative transition pathway. Decreasing the level of subsampling leads to reduced conformational diversity, as observed in the Abl1 and GMCSF examples. Interestingly, at low subsampling levels (512:1024, for instance), the CCR5 predictions still strongly sample both tested states, but an intermediate conformation which is significantly closer to the inactive reference is predicted more often. This effect is similar to the one observed by del Alamo and collaborators in their predictions of different conformations of CCR5 [14], and again reinforces the goldilocks principle of choosing subsampling conditions for predicting the relative populations of alternative states of a given system (that is, identifying the subsampling parameters that minimize the prediction of unfolded/nonphysical states while maximizing conformational diversity along a path defined by putative states as endpoints).

Finally, we hypothesize that our approach successfully predicts CCR5 in its active state (similar to PDB ID 7F1Qr) while previous subsampling methods fell short due primarily to the choice of using a deep MSA built with jackhmmer instead of mmseqs2. Recent works have shown that MSA depth and coverage directly affect AlphaFold2's accuracy [17], which is in line with our observations of how these factors impacted the CCR5 predictions.

LmrP

Multidrug transporters such as LmrP and LAT1 shift between two major states in their transport cycles, the outward-facing (OF) and the inward-facing (IF) conformations [18, 19]. In the CASP14 challenge, AlphaFold predicted LmrP with the highest confidence in the inward-facing conformation [20]. This is intriguing because previous studies have found that LmrP predominantly occupies the outward-facing conformation [19], and although there is a PDB structure of LmrP in the outward-facing conformation (PDB ID 6T1Za), it was published in 2020 and thus was not included in AlphaFold's training dataset [21]. Given this, the field's leading hypothesis for the preferential prediction of an alternate state of LmrP by AlphaFold was that other transporters in the IF conformation were present in the AlphaFold training dataset, leading to bias towards the prediction of LmrP in the IF conformation [14, 20].

In stark contrast to previous studies that predicted the structure of LmrP with AlphaFold [20], and as a direct refutation of the training bias hypothesis, our approach successfully predicts LmrP more frequently in the most stable state (OF) in certain subsampling conditions. These predictions occur in subsampling values below 64:128, after which the conformational preference is shifted and the IF state is predicted more often (Supplementary Figure 17).

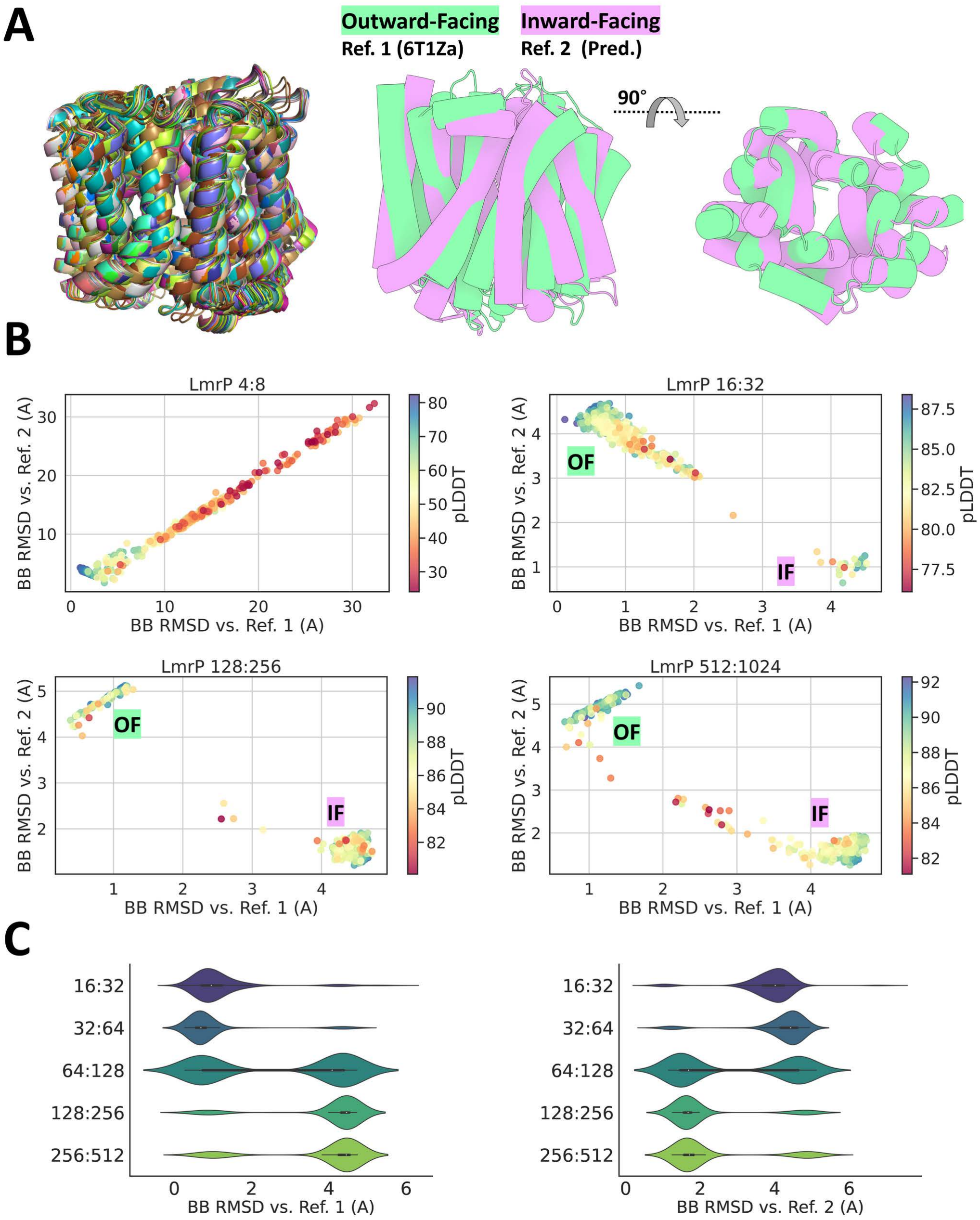

Supplementary Figure 17: Predictions for the LmrP system using the subsampled AF2 methodology described in this study. (A) (left) Structural models obtained from the prediction method, aligned to the top-ranked prediction by pLDDT (AF2's confidence metric) and overlaid on top of each other in different colors; (middle) Rendering of the conformational references used to summarize the prediction results in the backbone RMSD vs. references scatterplots. Each structure is colored according to its accompanying label; and (right) alternate view of the structural references. (B) Bidimensional projection of four sample prediction results, comparing the similarity of each prediction to either Ref. 1 (outward-facing, PDB ID 6T1Za) or Ref. 2 (inward-facing, AF2 prediction) by a backbone RMSD metric. Predictions are colored by average pLDDT, which is a metric for AlphaFold2's confidence in the resulting model. (C) Distribution of backbone RMSD values vs. each reference for each subsampling condition tested.

This prediction preference shift suggests that, in some cases, the selection of the appropriate subsampling conditions without prior knowledge is non-trivial. It is not within the scope of this study to resolve a one-size-fits-all approach for selecting subsampling conditions, but a few observations could form a general outline for further studies seeking to find a common heuristic toward that goal. As an example, we observed that subsampling levels that led to incorrect relative state populations (such as 128:256 and 512:1024) also led to the prediction of a significant number of conformations that did not closely map to the IF to OF putative pathway. The subsampling condition that mostly mirrored experimentally resolved conformational state populations (16:32) had very few predictions outside of that diagonal. This points towards a potential parameter for further evaluating subsampling conditions if the goal is to quantify relative state populations without any prior knowledge of the system.

Finally, we hypothesize that our approach successfully predicts LmrP in its OF state more frequently while previous subsampling methods failed due primarily to the choice of using a deep MSA built with jackhmmer instead of mmseqs2. Recent studies have shown that MSA depth and coverage directly affect AlphaFold2's accuracy [17], which is in line with our observations for how these factors impacted the LMRP and CCR5 predictions. Notably, predictions of LmrP with an MSA built from mmseqs2 only have the IF state as the most populated conformation regardless of subsampling level (Supplementary Figure 19), which we know to be inaccurate.

Effects of MSA Depth and Content

Considering the contrasting results obtained from the predictions made from multiple sequence alignments (MSAs) built from either the jackhmmer or mmseqs2 method, we sought to explore how MSA depth and content affected subsampled's AF2 ability to predict alternative conformations and relative state populations. The rationale for this test stems from the fact that jackhmmer frequently assembles significantly deeper MSAs than mmseqs2, due to differences in the queried datasets (jackhmmer searches UniRef90, smallbfd, and mgnify, while mmseqs2 searches UniRef100, PDB70, and an environmental sequence dataset) and due to mmseqs2 including an early stop heuristic to minimize the search space after a threshold of sequences is found [22].

Initially, we evaluated how wild-type Abl1 kinase core ensembles varied between predictions made with either the MSA built with jackhmmer (n = 614,759 sequences) or with mmseqs2 (n = 30,502 sequences). As an important control, we also evaluated predictions generated with a modified jackhmmer MSA, truncated at n = 30,502 sequences, in order to isolate the potential contributions of MSA composition beyond just depth. Importantly, this truncated jackhmmer MSA has the same number of sequences as the mmseqs2 MSA, but the sequences in the former are significantly more similar to each other than in the latter. As an additional control, we also made predictions with just the Abl1 kinase core sequence alone, obliviating any coevolutionary signal. The results of this analysis are summarized in Supplementary Figure 18.

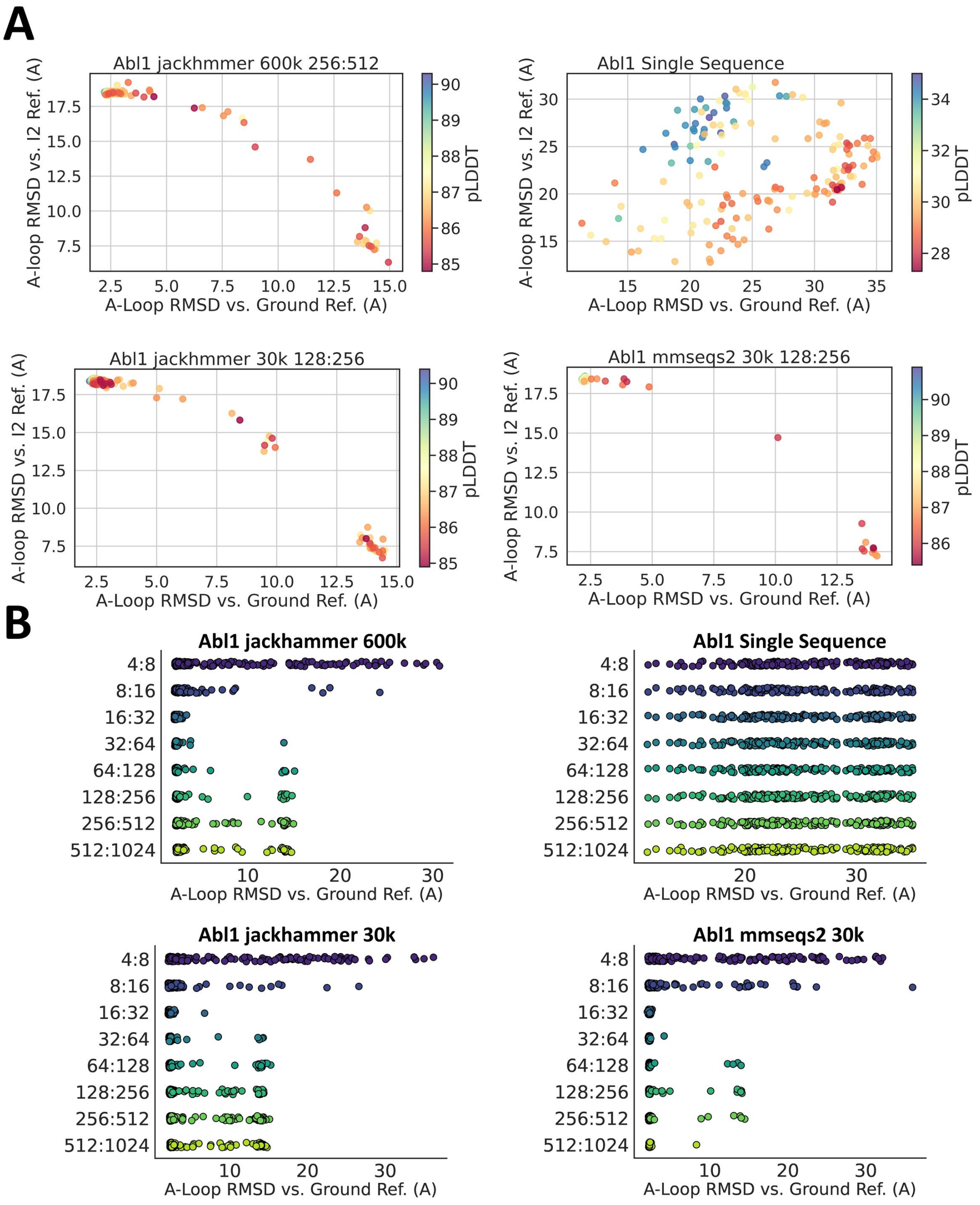

Supplementary Figure 18: Effects of MSA length and composition in AlphaFold2's capacity for predicting different conformations of the Abl1 kinase domain. (A) Bidimensional projection of prediction ensembles for the Abl1 kinase core in subsampling conditions 256:512 using different MSA lengths and compositions, summarized by the backbone RMSD of the A-Loop vs. the Ground (PDB ID 6XR6) or the I2 (PDB ID 6XRG) reference. Points are colored by pLDDT, which is a metric of AF2's confidence in the prediction. (B) Results for each prediction ensemble in different subsampling conditions ranging from 4:8 to 512:1024 with different MSA lengths and compositions, summarized as the backbone RMSD of the A-Loop of each prediction vs. the ground reference (PDB ID 6XR6). In both A and B, each dot represents a single prediction (n = 480).

Crucially, the ensemble resulting from the single sequence prediction leads to mostly unfolded structures that are not similar to the known organization of the Abl1 kinase core (or of any kinase core). This

suggests that, in the absence of templates, the presence of a coevolutionary signal from the input MSA is essential for accurately predicting kinase core conformations. In line with previous observations for the CCR5 and LmrP examples, the predictions using the mmseqs2 MSA as input led to considerably fewer intermediate conformation predictions for the Abl1 kinase core than those from the jackhmmer MSA. Interestingly, the truncated jackhammer MSA designed to be the same depth as the mmseqs2 MSA still led to considerably more conformations along the Ground to I2 path in Abl1 kinase core predictions. These results match recent studies that found that MSA depth leads to increased accuracy in AF2 predictions [17], while also recapitulating previous results that found that MSA entropy (that is, the average distance between pairs of sequences) also plays a significant role. Although it is within the scope of this study to answer why this is the case, we hypothesize that MSAs with lower entropy cause AF2 to more easily distill the coevolutionary signal pertaining to conformations that would otherwise be lost in MSAs with larger distances between sequences.

Considering the above and the observation that our subsampled AF2 approach using MSAs from jackhmmer succeeded at sampling challenges that were not met by previous studies using MSAs from mmseqs2, we repeated the CCR5 and LmrP predictions with MSAs from mmseqs2 and contrasted the results with our previously discussed prediction ensembles (generated with the jackhmmer MSAs). The results of this analysis are summarized in Supplementary Figure 19.

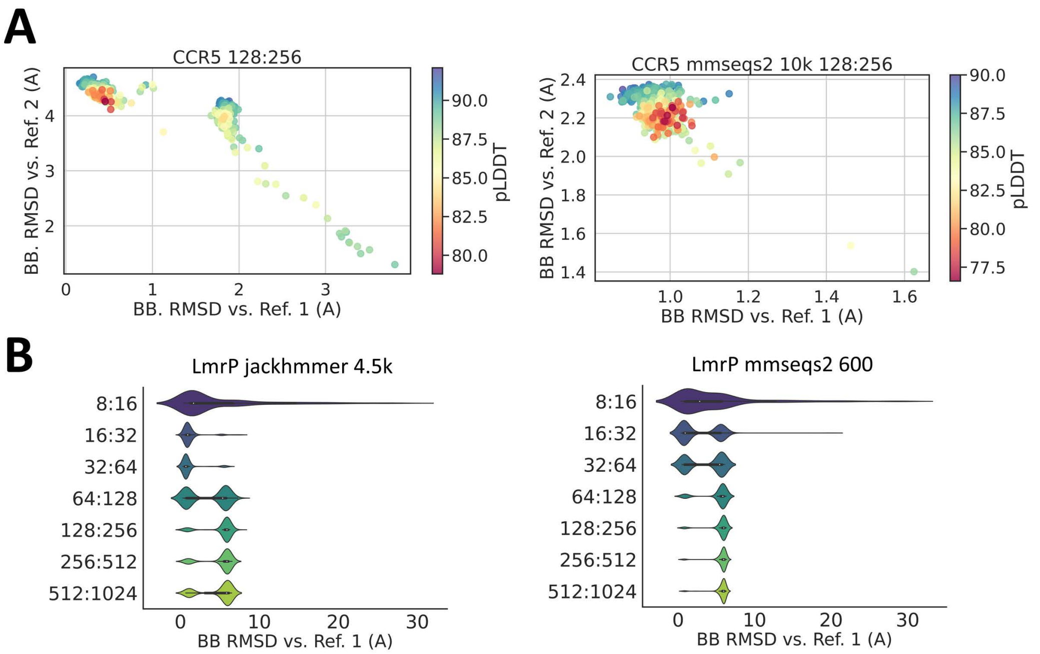

Supplementary Figure 19: Effects of MSA length and composition in AlphaFold2's capacity for predicting different conformations of CCR5 and LmrP. (A) Bidimensional projection of results for ensembles of CCR5 predictions generated with either the MSA from jackhmmer or from mmseqs2. Results are summarized according to a backbone RMSD metric vs. either the inactive state (Ref. 1, PDB ID 5UIWa), or the active state (Ref. 2, PDB ID 7F1Qr). (B) Distribution of LmrP predictions according to a backbone RMSD metric vs. the outward-facing conformation (Ref. 1, PDB ID 6T1Za). Ensembles were predicted from an MSA stemming from either jackhmmer (left) or mmseqs2 (right).

In the CCR5 example, predictions by del Alamo and collaborators did not lead to structures that significantly diverged from the conformation present in the AlphaFold training set [14]. These results are

replicated by our predictions with the mmseqs2 MSA (n = 10,066 sequences), where the vast majority of predictions in the ensemble represent the inactive form, which is present in the AF training set (inactive. PDB ID 5UIWa). As previously discussed in Supplementary Figure 16 and Supplementary Figure 19, our predictions with the MSA built from jackhmmer (n = 62,583 sequences) frequently populate the alternative state (active, PDB ID 7F1Qr, not present in the AF training set) and intermediate conformations between both states.

Additionally, both del Alamo and collaborators and the original implementation of AlphaFold for the CASP14 challenge found LmrP to be predicted more frequently in its inward-facing conformation [14, 20], despite the outward-facing conformation being the most frequently populated according to experimental data [19]. In Supplementary Figure 19, we show that predictions with the MSA from mmseqs2 (n = 628 sequences) lead to ensembles where the inward-facing conformation of LmrP is predicted in either similar frequencies to the outward-facing conformation, or exponentially more frequently. This is in stark contrast to the previously discussed predictions created from the jackhmmer MSA (n = 4,724 sequences), in which the outward-facing conformation is correctly predicted as the dominant state in certain subsampling conditions.

All in all, these results highlight the importance of considering MSA depth and entropy when seeking to predict the different conformational states of proteins and their relative state populations and should pave the way for future studies seeking to better understand what specific MSA elements are the most important for conformational preference in AF2 predictions.

LAT1

LAT1 is another transporter that converts between the inward-facing and outward-facing configurations [23], and was also tested in previous subsampling AF2 studies [14]. Contrary to CCR5, previous studies were successful in predicting both major conformations of LAT1 with AF2 [14]. To see how our approach fares at replicating the above results considering the contrasting results we obtained for CCR5 and LmrP, we made predictions for LAT1 using the previously described alternative subsampling conditions (4:8 to 1024:2048 max_seq:extra_seq, 480 individual predictions - 96 seeds * five models) and analyzed for the presence of both IF and OF conformations and putative in-between states. The results of these predictions are described in Supplementary Figure 20.

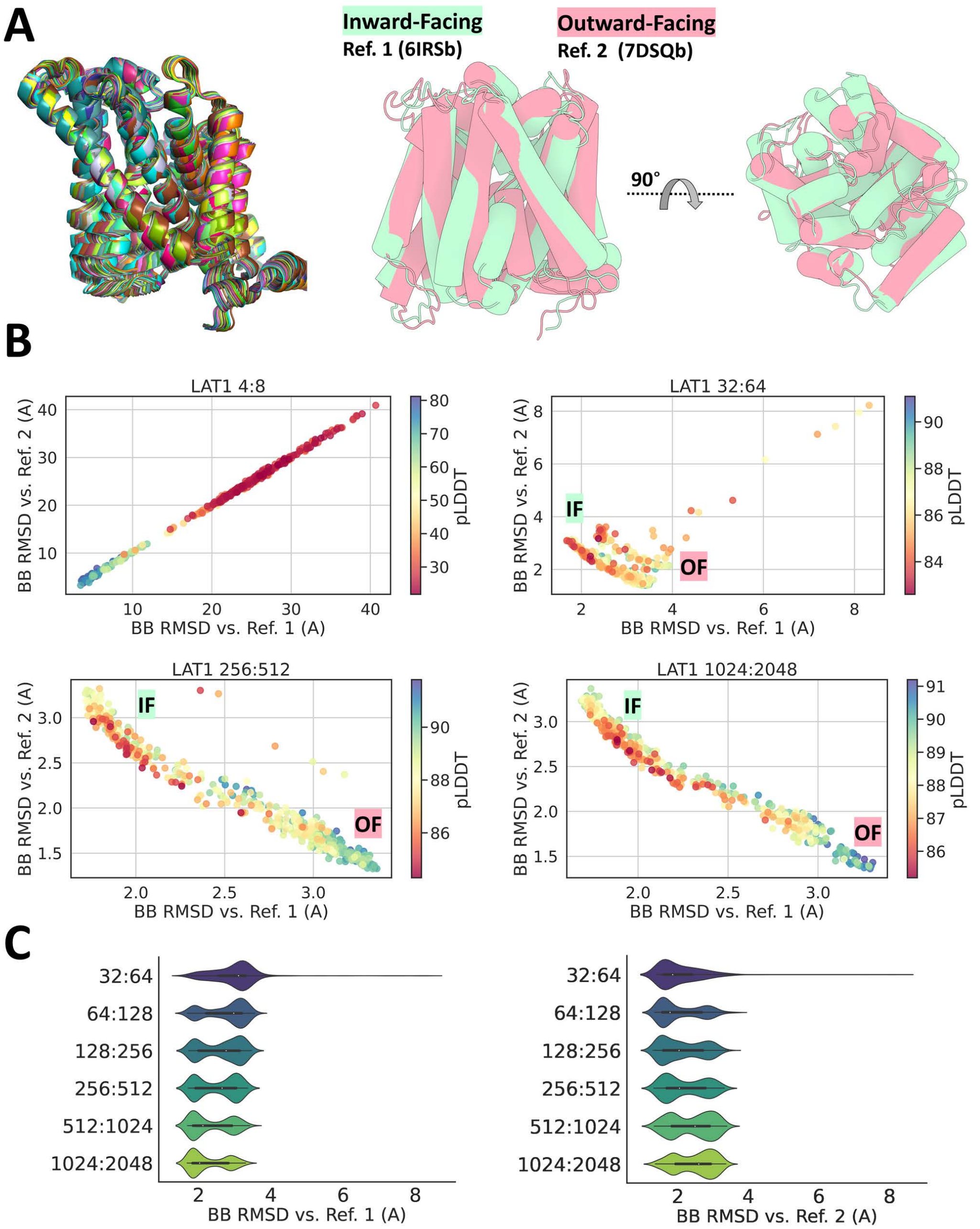

Supplementary Figure 20: Predictions for the LAT1 system using the subsampled AF2 methodology described in this study. (A) (left) Structural models obtained from the prediction method, aligned to the top-ranked prediction by pLDDT (AF2's confidence metric) and overlaid on top of each other in different colors; (middle) Rendering of the conformational references used to summarize the prediction results in the backbone RMSD vs. references scatterplots. Each structure is colored according to its accompanying label; and (right) alternate view of the structural references. (B) Bidimensional projection of four sample prediction results, comparing the similarity of each prediction to either Ref. 1 (inward-facing, PDB ID 6IRSb) or Ref. 2 (outward-facing, 7DSQb) by a backbone RMSD metric. Predictions are colored by average pLDDT, which is a metric for AlphaFold2's confidence in the resulting model. (C) Distribution of backbone RMSD values vs. each reference for each subsampling condition tested.

Notably, our predictions closely resemble those obtained by del Alamo and collaborators in terms of the distribution between conformations at different subsampling conditions [14], with lower subsampling conditions such as 256:512 and above leading to predictions primarily of the IF state.

Calmodulin

Calmodulin is a 16.7 kDa (148 AA), highly conserved calcium-binding protein composed of two symmetrical terminal globular domains connected by a flexible linker [24]. Each terminal domain contains a pair of EF-hand motifs, for a total of four calcium binding sites [25]. In the absence of calcium and/or in the presence of binders, Calmodulin assumes a collapsed and compact form, with the central linker disordered [26, 27]. The apo version of the protein becomes highly organized upon calcium saturation, and the central linker forms a mostly stable helix that converts between a fully extended and a bent conformation in solution [27]. Importantly, E84 deletions in Calmodulin are known to change the propensity for the formation of the extended form of calcium-saturated apo Calmodulin in solution [28], and the M124L mutation has similar effects to E84K in biochemical assays [29].

Considering the above, we sought to test how our subsampled AF2 approach fares at predicting the interesting intrinsic dynamics of calcium-saturated apo calmodulin, as well as the effects of the two point mutations (E84K and M124L) suspected to alter its conformational equilibrium [28, 29]. To do so, we first predicted the structure of chicken Calmodulin using subsampled AF2 with different subsampling conditions ranging from 4:8 to 1024:2048 (max_seq:extra_seq), with 480 individual predictions for each condition (96 seeds times 5 models) and evaluated the resulting ensembles (Supplementary Figure 21).

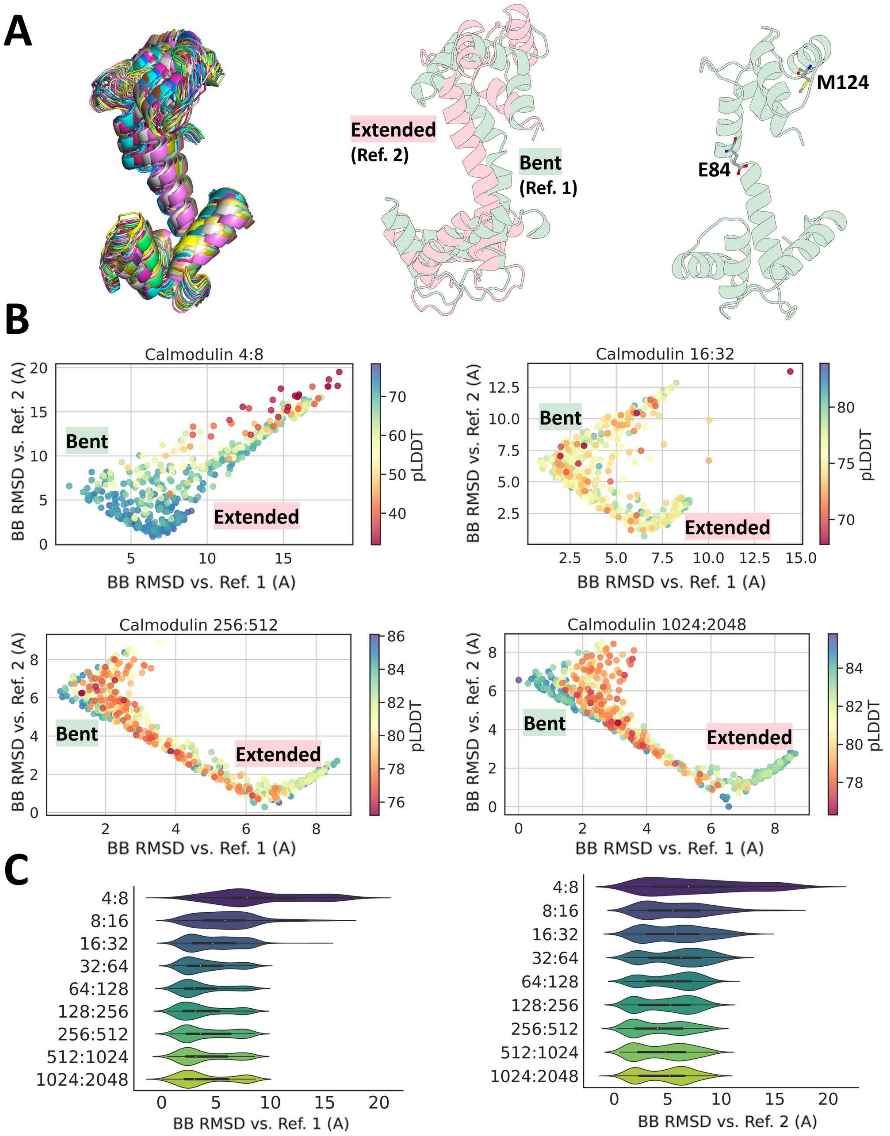

Supplementary Figure 21: Predictions for the Calmodulin system using the subsampled AF2 methodology described in this study. (A) (left) Structural models obtained from the prediction method, aligned to the top-ranked prediction by pLDDT (AF2's confidence metric) and overlaid on top of each other in different colors; (middle) Rendering of the conformational references used to summarize the prediction results in the RMSD vs. references scatterplots. Each structure is colored according to its accompanying label; and (right) Positions of residues suspected to affect relative state populations when mutated. (B) Bidimensional projection of four sample prediction results, comparing the similarity of each prediction to either Ref. 1 (bent central linker, PDB ID 4BW7b) or Ref. 2 (extended central linker, PDB ID 4BW8b) using a backbone RMSD metric. Predictions are colored by average pLDDT, which is a metric for AlphaFold2's confidence in the resulting model. (C) Distribution of backbone RMSD values vs. each reference for each subsampling condition tested (4:8 and 8:16 are omitted due to a high frequency of unfolded predictions, which would warp the X axis).

Analysis revealed that the vast majority of the predicted structures adopt the ordered conformation (Supplementary Figure 21A). Although AF2 does not allow for the inclusion of ions in the modeling process, this preference towards the ordered conformation might be due to training set composition biases.

Importantly, for most of the subsampling conditions, the ensembles presented a bimodal distribution of conformations, with the first mode representing the ordered conformation of Calmodulin with the central linker bent, consistent with previous studies that found that calcium-saturated chicken Calmodulin assumes this conformation in solution [30]. The other mode, significantly less populated, corresponds to the ordered Calmodulin conformation with the fully extended central linker [28].

Besides the identification of two significantly populated conformations of calcium-saturated apo chicken Calmodulin in the wild-type prediction, subsampling conditions above 8:16 also led to the prediction of a range of intermediate conformations between each stable state (Supplementary Figure 21B). Crucially, the intermediate conformations appear to cover most of the range between the bent and extended states.

Considering the success of subsampled AF2's approach in predicting the two main states of ordered chicken Calmodulin, we sought to test if our heuristic could also correctly predict the suspected effects of the E84K and M124L mutations. For this comparison, we chose the 256:512 subsampling conditions because they led to the best coverage of the putative path between the two main states in the wild-type predictions.

As seen in Supplementary Figure 22, our approach predicts that the E84K mutation increases the propensity for forming the extended linker, which is a phenotype similar to E84 deletions [28], as that state's relative population is significantly increased in our E84K predictions. Additionally, the M124L mutation has similar effects to E84K in biochemical assays and is hypothesized to also affect linker conformation [29]. This potential similarity is captured by the subsampled AF2 predictions for the M124L mutant, which led to a reduced population of the bent conformation, although not as drastic as the E84K mutation.

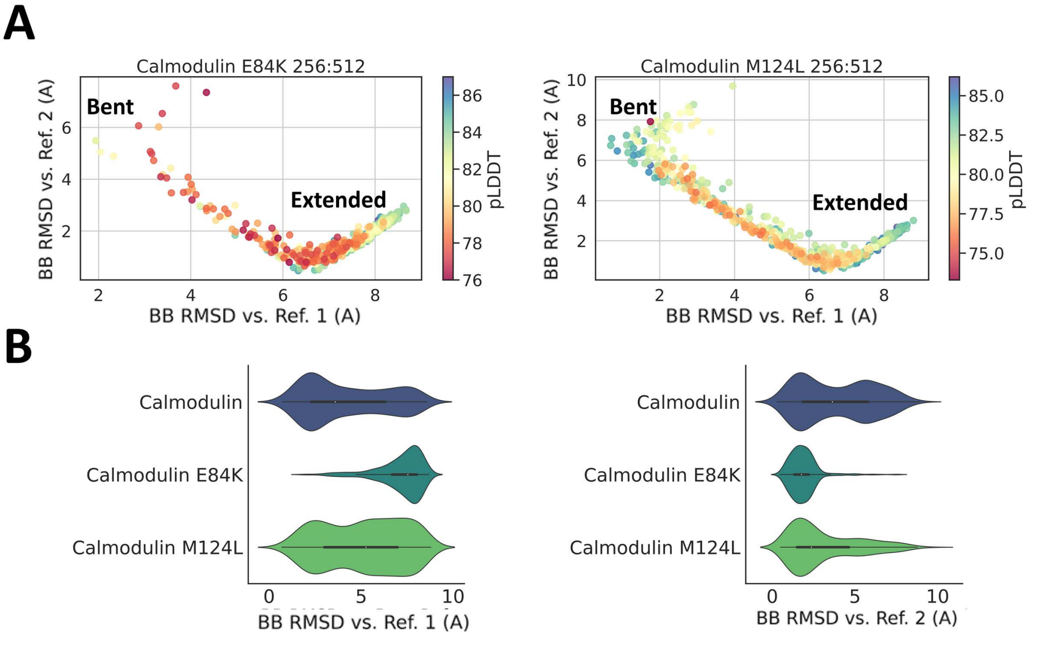

Supplementary Figure 22: Predictions for Calmodulin mutants. (A) Bidimensional projection of predictions for the (left) E84K mutant with 256:512 subsampling conditions and (right) M124L mutant with 256:512 subsampling conditions, comparing the similarity of each prediction to either Ref. 1 (bent central linker, PDB ID 4BW7b) or Ref. 2 (extended central linker, PDB ID 4BW8a). (B) Distribution of backbone RMSD values vs. each reference for the wild-type reference and for the tested mutants in the 256:512 subsampling conditions.

In summary, our subsampled AF2 approach correctly identified the two main states of calcium-saturated chicken Calmodulin. The resulting predictions are distributed in a manner that correlates with experimentally determined conformational preferences. Finally, our approach also correctly predicted the effects of two mutations suspected to decrease the stability of the bent linker state, by leading to ensembles in which the extended state is predicted more frequently.

Fyn-SH3 Triple Mutant

The Src-homology 3 (SH3) is a protein domain composed of approximately 65 amino acids [31]. It is found in a large number of eukaryotic proteins related to signal transduction and is functionally important for protein/protein interactions [31, 32]. Fyn, a kinase of the Src family, contains an SH3 domain (Fyn-SH3) that is crucial for regulating kinase activity [33, 34]. Fyn-SH3 has been previously used as a model for studying protein folding [35–38], and information about the relative population of different states is abundant [39]. Further, substitutions in Fyn-SH3 such as the triple mutant A39V+N53P+V55L are known to cause it to interact strongly with other copies of itself, leading to aggregation [39]. Importantly, these mutations lead to the aggregation phenotype by disrupting the order of the C-terminus of Fyn-SH3, which preferentially forms a stable beta-sheet [39]. In the mutant proteins, the C-terminus of Fyn-SH3 is significantly less stable during the folding process, exposing the aggregation-prone amino-terminal beta strand [39].

The interesting dynamics of SH3 domains and the extensive literature pertaining to altered conformational equilibriums in response to mutations make Fyn-SH3 an excellent challenge for our subsampled AF2 method. We started by making predictions of the triple mutant form of Fyn-SH3 (residues 7 to 63, based on PDB ID 2LP5a) using different subsampling levels, ranging from 4:8 to 1024:2048, with a sample size of 480 predictions per ensemble (96 seeds * 5 models). The results of this are presented in Supplementary Figure 23.

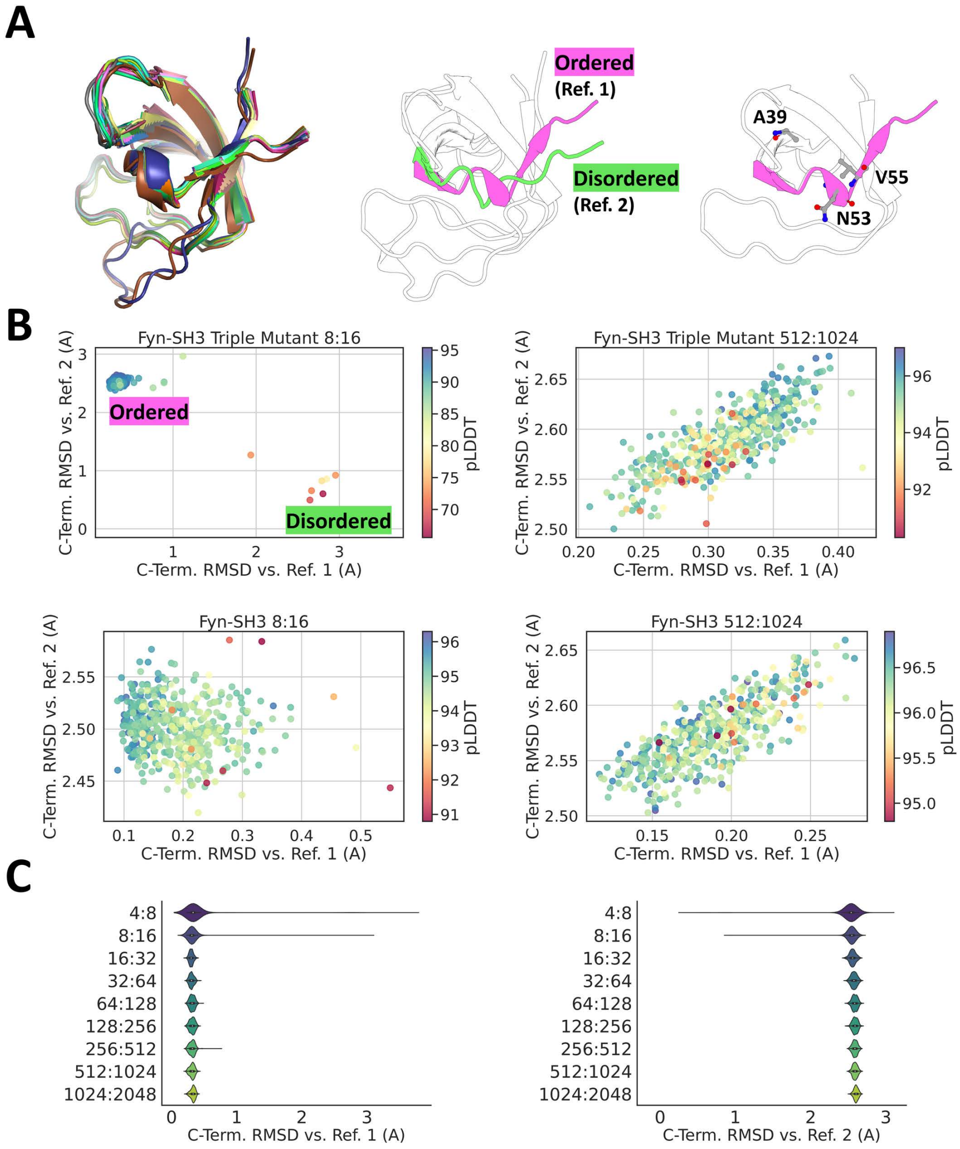

Supplementary Figure 23: Predictions for the Fyn-SH3 triple mutant/wild-type system using the subsampled AF2 methodology described in this study. (A) (left) Structural models obtained from the prediction method, aligned to the top-ranked prediction by pLDDT (AF2's confidence metric) and overlaid on top of each other in different colors; (middle) Rendering of the conformational references used to summarize the prediction results in the C-terminus backbone RMSD vs. references scatterplots. Each structure is colored according to its accompanying label; and (right) position of residues known to affect relative state populations when mutated. (B) Bidimensional projection of two sample prediction results for triple mutant Fyn-SH3 (top) or to wild-type Fyn-SH3 (bottom) comparing the similarity of each prediction to either Ref. 1 (ordered C-terminus, chosen from the AF2 prediction ensemble) or Ref. 2 (disordered C-terminus, chosen from the AF2 prediction ensemble) by a C-terminus backbone RMSD metric. Predictions are colored by average pLDDT, which is a metric of AlphaFold2's confidence in the resulting model. (C) Distribution of C-terminus backbone RMSD values vs. each reference for each subsampling condition tested for triple mutant Fyn-SH3.

The decision to start with the triple mutant is rooted in the fact that the conformation with the disor-

dered C-terminus is stabilized in the triple mutant, with a population of 2%, and has not been detected in the wild-type.

Surprisingly, only the subsampling conditions 4:8 and 8:16 (max_seq:extra_seq) led to the detection of the Fyn-SH3 state containing a disordered C-terminus. This might stem from the very low population of this state even in the triple mutant, which has been measured experimentally as about 2% [39], and/or from potential biases stemming from training set composition (most SH3 structures in the PDB have an ordered C-terminus). Additionally, only a handful of predictions were found to be in the alternative conformation (six in the 8:16 subsampling conditions, from a total of 480 predictions, or 1.25% of the total), and no significant coverage of intermediate conformations between ordered and disordered C-terminus was found in any subsampling condition. These results hint at a resolution limitation of subsampled AF2, which did not perform well at predicting intermediate conformations in this example, and required extreme subsampling to detect the disordered C-terminus conformation of triple mutant Fyn-SH3.

Next, we repeated our prediction heuristic for wild-type Fyn-SH3, which led to no predictions of conformations with the disordered C-terminus, regardless of subsampling level (Supplementary Figure 23B). This is not unexpected, as this conformation in wild-type Fyn-SH3 is present in presumably undetectable levels (if at all), and the A39V+N53P+V55L mutations are required to stabilize it sufficiently for detection. Considering the potential resolution limitations described above and the presumably extremely low population of this conformation in wild-type Fyn-SH3, it is unsurprising that AF2 was not able to predict it, even with extreme subsampling levels.

AlkB

Alkylation B (AlkB) is a bacterial protein that is involved in the adaptive response by reversing alkylation damage from single-stranded DNA [40]. In solution, AlkB occupies two predominant conformations, open and closed, with the closed conformation being significantly more stable in the presence of zinc and of the co-substrate 2OG. [41].

Given the presence of two distinct conformational states in AlkB and literature pertaining to their relative state populations, we sought to measure how our subsampled AF2 approach fared at predicting the two major states of AlkB in the right proportion. As with the previous examples, we made AlkB predictions with subsampling conditions ranging from 4:8 to 1024:2048, with 480 individual predictions per condition (96 seeds time five models). The results of the AlkB predictions with subsampled AF2 are described in Supplementary Figure 24.

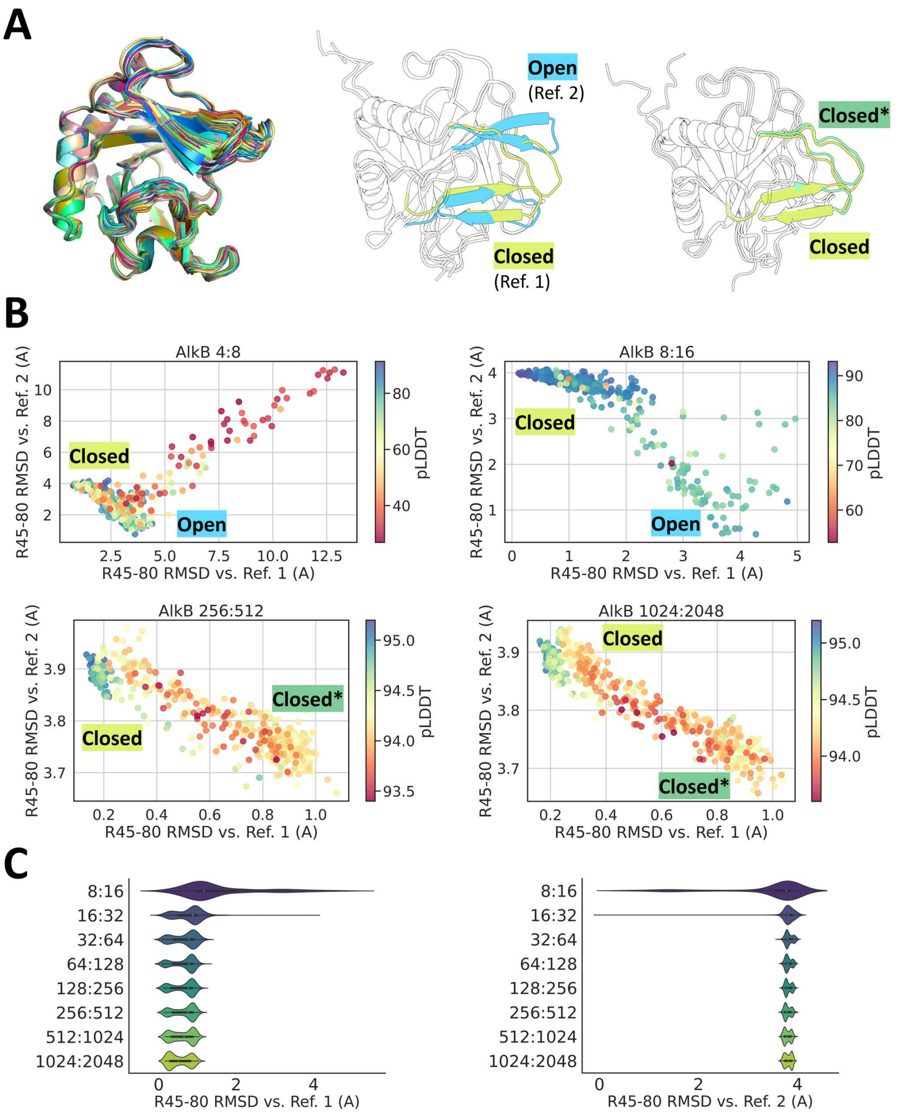

Supplementary Figure 24: Predictions for the AlkB system using the subsampled AF2 methodology described in this study. (A) (left) Structural models obtained from the prediction method, aligned to the top-ranked prediction by pLDDT (AF2's confidence metric) and overlaid on top of each other in different colors; (middle) Rendering of the conformational references used to summarize the prediction results in the binding site backbone RMSD vs. references scatterplots. Each structure is colored according to its accompanying label; and (right) Comparison between the closed conformation of AlkB and a slightly open conformation that is predicted frequently with certain subsampling conditions. (B) Bidimensional projection of four sample prediction results, comparing the similarity of each prediction to either Ref. 1 (closed binding site, PDB ID 3I49a) or Ref. 2 (open binding site, AF2 prediction) by a C-terminus backbone RMSD metric. Predictions are colored by average pLDDT, which is a metric of AlphaFold2's confidence in the resulting model. (C) Distribution of binding site backbone RMSD values vs. each reference for each subsampling condition tested (subsampling condition 4:8 omitted from the plot to avoid distortion of the X axis).

Importantly, our subsampled AF2 approach correctly captures the open and closed conformations of AlkB with certain subsampling conditions such as 8:16, with strong coverage of intermediate confor-

mations in the putative transition. The closed conformation is predicted far more frequently than the open conformation, which is interesting as that conformation only becomes dominant upon the binding of zinc and of the co-substrate 2OG [40], indicating a potential bias in AF2's predictions that cause the method to preferentially predict the bound form of the AlkB even in the absence of explicit substrate or ion coordination. Interestingly and similar to the CCR5 example, reducing subsampling levels leads to a reduction in conformational diversity, to the point that the proper "open" conformation is not predicted after max_seq:extra_seq values of 16:32. The ensembles resulting from predictions above this threshold are still strongly bimodal, but the conformational change between the ground state and the alternative state is very minute, although it is still on the pathway towards the open conformation.

Ultimately, our subsampled AF2 approach was successful in predicting both predominant conformations of AlkB, although the proportions of each prediction did not match what is expected in the literature for the apo form of the enzyme. The observed effect of loss of conformational diversity at lower subsampling levels is similar to the one observed in the Abl1, and GMCSF examples, highlighting the importance of choosing appropriate subsampling conditions for predicting the alternative states of a given system.

Aurora Kinase A

Aurora A is a serine/threonine kinase involved in crucial processes during mitosis and meiosis, playing a central role in cell proliferation [42]. As with Abl1, Src, and other kinases, Aurora A can shift between active and inactive forms through a conformational change know as the DFG flip pathway [43]. Improper regulation of Aurora A kinase activity can be remediated with kinase inhibitors, although that can be challenging without causing off-site effects [44]. To circumvent this problem, inhibitors selective for Aurora A kinase have been discovered and/or designed, including the inhibitor known as MLN8054 [45, 46].

Interestingly, MLN8054 stands out from other kinase inhibitors because it is thought to induce and bind to the "DFG-up" conformation in Aurora A [47]. Notably, this conformation is theorized to be an intermediate conformation in the kinase inactivation pathway (DFG flip) that is presumably at too low occupancy to be detected with NMR methods in other kinases such as Abl1 [1]. Since MLN805 preferentially binds to the DFG-up conformation, and MLN8054 is highly selective towards Aurora Kinase A, we hypothesize that the intermediate conformations in the inactivation pathway might be considerably more stable in Aurora Kinase A, and that our subsampled AF2 approach could detect this change in stability. This hypothesis is supported by the observation that Imatinib, which binds to the DFG-out conformation of kinases, is highly selective towards Abl1, which occupies the DFG-out conformation significantly more often than Src [48], a phenotype that is captured by subsampled AF2.

To test if the Aurora A kinase domain occupies intermediate conformations in the inactivation pathway more frequently than other kinases such as Abl1, we applied our subsampled AF2 protocol with the Aurora A kinase core, using AF2 to make predictions with subsampling parameters ranging from 4:8 to 1024:2048 (max_seq:extra_seq), totaling 480 predictions per condition (96 independent seeds times five models). The results of these predictions are summarized in Supplementary Figure 25.

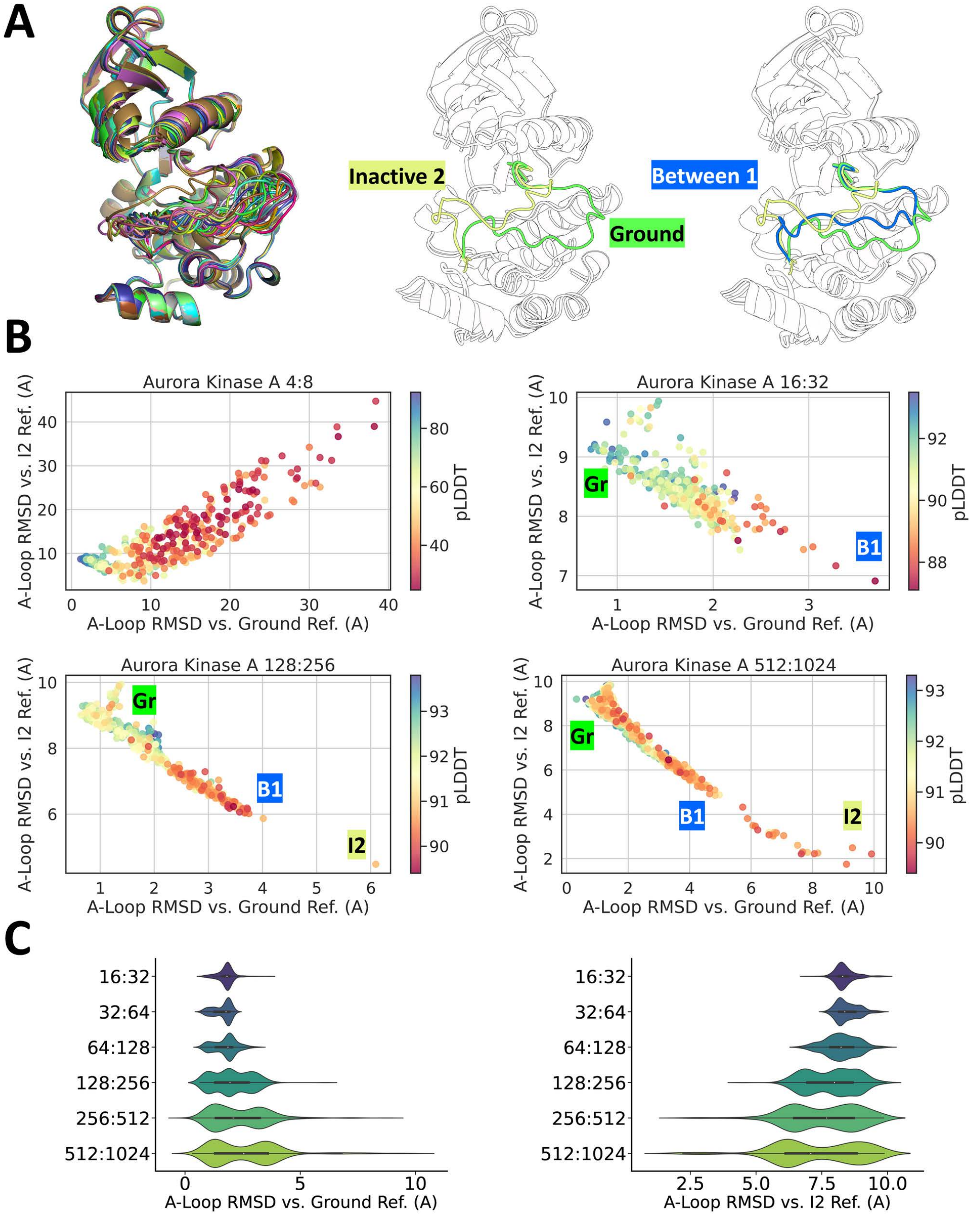

Supplementary Figure 25: Predictions for the Aurora Kinase A system using the subsampled AF2 methodology described in this study. (A) (left) Structural models obtained from the prediction method, aligned to the top-ranked prediction by pLDDT (AF2's confidence metric) and overlaid on top of each other in different colors; (middle) Rendering of the conformational references used to summarize the prediction results in the A-Loop backbone RMSD vs. references scatterplots. Each structure is colored according to its accompanying label; and (right) Comparison between the ground-like state, I2-like state, and a putative intermediate conformation that is significantly enriched in the Aurora Kinase A predictions. (B) Bidimensional projection of four sample prediction results, comparing the similarity of each prediction to either Ref. 1 (ground-like) or Ref. 2 (i2-like) by a A-Loop backbone RMSD metric. Predictions are colored by average pLDDT, which is a metric of AlphaFold2's confidence in the resulting model. (C) Distribution of A-Loop backbone RMSD values vs. each reference for each subsampling condition tested (subsampling conditions 4:8 through 8:16 omitted from the plot to avoid distortion of the X axis).

Curiously, our Aurora Kinase A prediction ensembles differ from the Abl1 and Src predictions in that the resulting RMSD distributions vs. known references (Ground or I2) is trimodal in certain subsampling conditions (such as 256:512 or 512:1024), with a putative intermediate conformation being predicted with similar frequencies than Ground-like conformations. We speculate that the enrichment of this intermediate conformation in Aurora Kinase A when compared to Abl1 or Src provides support to the hypothesis that intermediate states might be occupied more frequently in Aurora A.

Carbonic Anhydrase

Carbonic Anhydrase (CA) is an enzyme that helps maintain acid-base balance by catalyzing the interconversion between carbon dioxide and water and the dissociated ions of carbonic acid [49, 50]. We included CA in the analysis because its enzymatic domain is knotted and shows very little conformational mobility [50, 51], so it is a welcome control case to measure if our subsampled AF2 approach might be exaggerating the frequency and amplitude of conformational changes in proteins. For this, we repeated the experimental routine described for all of our previous systems, making predictions for subsampling conditions 4:8 to 1024:2048, with 480 individual predictions for each condition (96 seeds times five models) for human Carbonic Anhydrase VI [50]. The results for the CA predictions are described in Supplementary Figure 26.

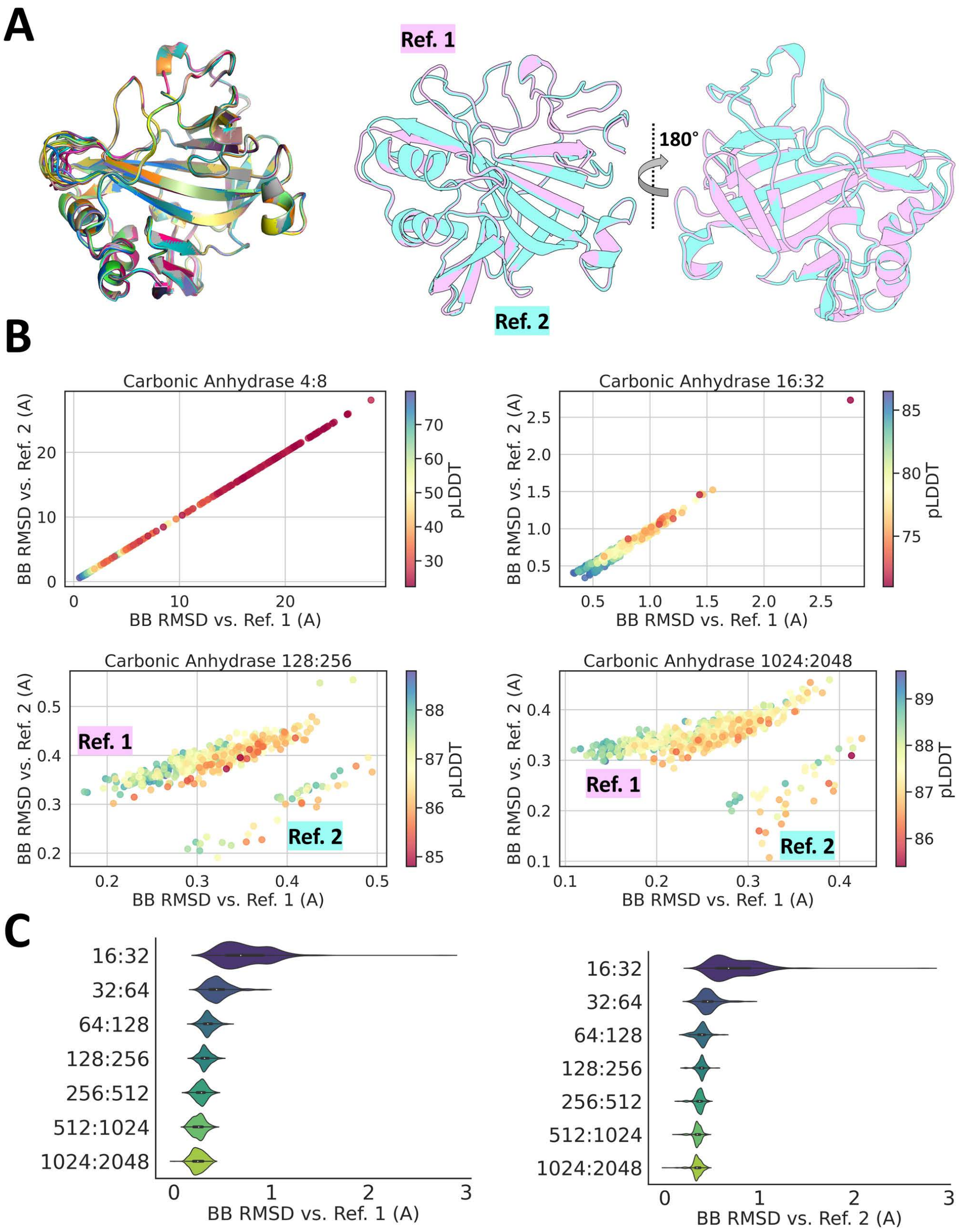

Supplementary Figure 26: Predictions for the human Carbonic Anhydrase VI system using the subsampled AF2 methodology described in this study. (A) (left) Structural models obtained from the prediction method, aligned to the top-ranked prediction by pLDDT (AF2's confidence metric) and overlaid on top of each other in different colors; (middle) Rendering of the conformational references used to summarize the predictions in the backbone RMSD vs. reference scatterplots. Each structure is colored according to its accompanying label; and (right) alternate view of the structural references. (B) Bidimensional projection of four sample predictions, comparing the similarity of each prediction to either Ref. 1 (human CA VI, AF2 prediction most similar to PDB ID 3FE4a) or Ref. 2 (bottom-ranked structure by pLDDT) by a backbone RMSD metric. Predictions are colored by average pLDDT, which is a metric of AlphaFold2's confidence in the resulting model. (C) Distribution of backbone RMSD values vs. each reference for each subsampling condition tested (subsampling conditions 4:8 and 8:16 are omitted from the plots to avoid distorting the X axis).

Notably, every subsampling condition above 16:32 led to predictions with extremely small conformational diversity for Carbonic Anhydrase, and conditions below that threshold led to predictions of mostly unfolded/misfolded structures that do not correspond to known conformational states of CA. As previously mentioned, the amplitude of conformational changes in CA across each predicted ensemble is minute and mostly provoked by the dynamics of a small flexible loop (residues 116-120) in CA VI. Further analysis and comparison with other prediction sets show that other structural elements of Carbonic Anhydrase, which have no other known conformational states, are very rigid across predictions.

These results are not unexpected and highlight the point previously illustrated by the distribution of RMSD values vs. ground or alternative states in previous examples, which is that subsampled AF2 with optimized subsampling parameters is correctly predicting conformational changes in domains known to change conformation or to be flexible, instead of randomly predicting dynamics across protein backbones.

As a positive control of random predictions of dynamics, we point to extreme subsampling conditions such as 4:8 in the CA and other examples, where the resulting ensemble is extremely diverse with many different conformations that are, to the best of our knowledge, not representative of actual states.

Additional Negative Controls

In addition to the Carbonic Anhydrase VI test, which is a protein with very little conformational mobility across most of its backbone, we also sought to test if subsampled AF2 was correctly predicting the rigidity of structural elements known to not be mobile even in proteins that undergo significant conformational changes.

For that test, we measured the backbone RMSD vs. the ground and alternative references from subsampled AF2 prediction ensembles of two structural elements known to be relatively immobile belonging to either the Abl1 kinase core or the AlkB enzyme. For the Abl1 kinase core, we chose residue range 419-434, as that forms a structural helix in the C-lobe that is seldom disrupted and not involved in the activation/inactivation pathway. For the AlKB test case, we chose the residue range 150-200, which forms half of the beta-sandwich in AlkB and is known to be stable and not involved in AlkB opening and closing. The results of this analysis, as well as comparisons with bona fide structural changes observed in other structural elements of these example proteins, are summarized in Supplementary Figure 27.

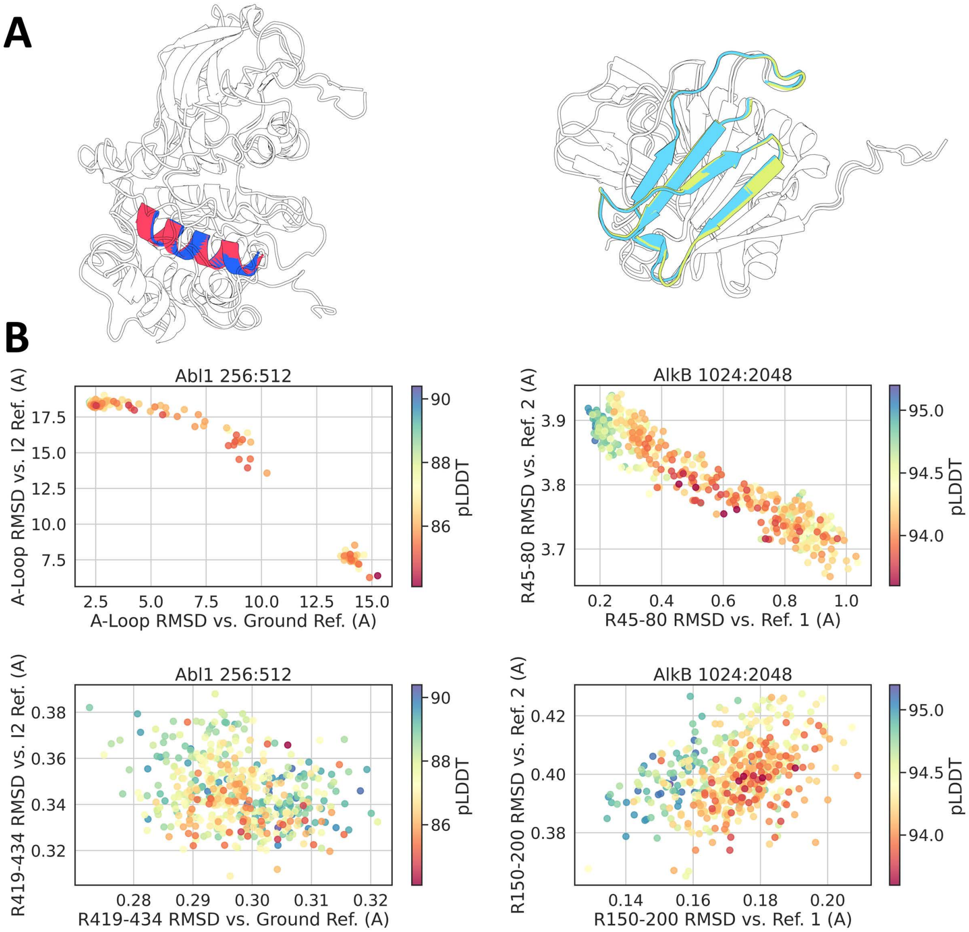

Supplementary Figure 27: Comparison of prediction results using subsampled AF2 for mobile and rigid structural elements of the Abl1 kinase core and of AlkB. (A) (left) Superposition of structural models of the Abl1 kinase core in the active or inactive conformations with residue range 419-434 colored in red in the active core, and in blue in the inactive core, (right) Superposition of structural models of the AlkB enzyme in the closed or open conformations with residue range 150-200 colored in yellow in the closed state, and in cyan in the open state (B) (left) Bidimensional projection of results from the Abl1 kinase core prediction ensemble with 256:512 subsampling parameters, comparing the backbone RMSD distribution vs. the inactive and vs. the active references for the mobile activation loop (top) or for the rigid helix formed by residues 419-434 (bottom); (right) Bidimensional projection of results from the AlkB prediction ensemble with 1024:2048 subsampling parameters, comparing the backbone RMSD distribution vs. the closed and vs. the open references for the mobile binding site (top) or for the rigid beta sheets formed by residues 150-200 (bottom).

Notably, within both prediction ensembles, the backbone RMSD vs. references for the rigid elements did not cross the 0.5 A threshold, as opposed to the known mobile elements that ranged up to 15 A in the case of Abl1. Additionally, the distribution of RMSDs for the rigid elements did not follow either the signature downwards diagonal (strong negative correlation) observed in predictions covering a conformational change, or the upwards diagonal (strong positive correlation) observed in predictions that diverge significantly from both references. Combined, these results suggest that AF2 is correctly predicting rigid structural elements to be rigid and mobile structural elements to be mobile in Abl1 and AlkB in the tested subsampling conditions.

Statistics of Measurements

In order to measure the significance of the differences observed in structural ensembles for the multiple wild-type vs. variants predictions generated in this study, we used a Kruskal-Wallis H-test between each Wild-Type/variant pair. A sample of the results of this analysis are summarized in Supplementary Table 4. Complete results are available in the GitHub repository used for data deposition in this study [52].

p_value	h_stat	trial	test	sample_size
1	0	GMCSF	R80-90 BB. RMSD vs. Ref.	480
2.99E-26	112.3543	GMCSF H15Y	R80-90 BB. RMSD vs. Ref.	480
4.18E-47	207.7826	GMCSF H15R	R80-90 BB. RMSD vs. Ref.	480
7.70E-27	115.0427	GMCSF H15N	R80-90 BB. RMSD vs. Ref.	480
1	0	Calmodulin	BB. RMSD vs. Ref.	480
8.78E-78	348.5452239	Calmodulin E84K	BB. RMSD vs. Ref.	480
1.63E-09	36.36993935	Calmodulin M124L	BB. RMSD vs. Ref.	480
1	0	Fyn SH3	C-Term, RMSD vs. Ref.	480
4.72E-22	93.20141568	Fyn SH3 A39V/N53P/V55L	C-Term. RMSD vs. Ref.	480
1	0	Abl1	A-Loop RMSD vs. Ref.	480
1.66E-12	49.84482421	Anc-AS	A-Loop RMSD vs. Ref.	480
1.88E-17	72.26142001	Src	A-Loop RMSD vs. Ref.	480
1.18E-11	46.00662103	Abl1 M290L + L301I	A-Loop RMSD vs. Ref.	480
0.34444905	0.893802102	Abl1 E255V + T315I	A-Loop RMSD vs. Ref.	480

Notably, most variants led to distributions of structural observables that are significantly different than the wild-type measurements, with the exception of a few Abl1 activating mutations for which statistical power was reduced.

Supplementary References

1. Xie, T., Saleh, T., Rossi, P. & Kalodimos, C. G. Conformational states dynamically populated by a kinase determine its function. *Science* **370** (Oct. 2020).
2. Finn, R. D., Clements, J. & Eddy, S. R. HMMER web server: interactive sequence similarity searching. *Nucleic Acids Research* **39,** W29–W37 (May 2011).
3. Suzek, B. E., Wang, Y., Huang, H., McGarvey, P. B. & and, C. H. W. UniRef clusters: a comprehensive and scalable alternative for improving sequence similarity searches. *Bioinformatics* **31,** 926–932 (Nov. 2014).
4. Steinegger, M. & Söding, J. Clustering huge protein sequence sets in linear time. *Nature Communications* **9** (June 2018).
5. Richardson, L. *et al.* MGnify: the microbiome sequence data analysis resource in 2023. *Nucleic Acids Research* **51,** D753–D759 (Dec. 2022).
6. Eastman, P. *et al.* OpenMM 7: Rapid development of high performance algorithms for molecular dynamics. *PLOS Computational Biology* **13** (ed Gentleman, R.) e1005659 (July 2017).
7. Lindorff-Larsen, K. *et al.* Improved side-chain torsion potentials for the Amber ff99SB protein force field. *Proteins: Structure, Function, and Bioinformatics* **78,** 1950–1958 (Mar. 2010).

8. Mark, P. & Nilsson, L. Structure and Dynamics of the TIP3P, SPC, and SPC/E Water Models at 298 K. *The Journal of Physical Chemistry A* **105,** 9954–9960 (Oct. 2001).

9. Hess, B., Bekker, H., Berendsen, H. J. C. & Fraaije, J. G. E. M. LINCS: A linear constraint solver for molecular simulations. *Journal of Computational Chemistry* **18,** 1463–1472 (Sept. 1997).

10. Bogetti, A. T. *et al.* A Suite of Advanced Tutorials for the WESTPA 2.0 Rare-Events Sampling Software [Article v2.0]. *Living Journal of Computational Molecular Science* **5** (2022).

11. Service, R. 'The game has changed.' AI triumphs at solving protein structures. *Science* (Nov. 2020).

12. Roney, J. P. & Ovchinnikov, S. State-of-the-Art Estimation of Protein Model Accuracy Using AlphaFold. *Physical Review Letters* **129** (Nov. 2022).

13. Walter, M. R. *et al.* Three-dimensional structure of recombinant human granulocyte-macrophage colony-stimulating factor. *Journal of Molecular Biology* **224,** 1075–1085 (Apr. 1992).

14. Del Alamo, D., Sala, D., Mchaourab, H. S. & Meiler, J. Sampling alternative conformational states of transporters and receptors with AlphaFold2. *eLife* **11** (eds Robertson, J. L., Swartz, K. J. & Robertson, J. L.) Publisher: eLife Sciences Publications, Ltd, e75751 (Mar. 2022).

15. Jiao, X. *et al.* Recent Advances Targeting CCR5 for Cancer and Its Role in Immuno-Oncology. *Cancer Research* **79,** 4801–4807 (Oct. 2019).

16. Zhang, H. *et al.* Structural basis for chemokine recognition and receptor activation of chemokine receptor CCR5. *Nature Communications* **12** (July 2021).

17. Lee, S. *et al.* Petascale Homology Search for Structure Prediction (July 2023).

18. Boudker, O. & Verdon, G. Structural perspectives on secondary active transporters. *Trends in Pharmacological Sciences* **31,** 418–426 (Sept. 2010).

19. Masureel, M. *et al.* Protonation drives the conformational switch in the multidrug transporter LmrP. *Nature Chemical Biology* **10,** 149–155 (Dec. 2013).

20. Del Alamo, D., Govaerts, C. & Mchaourab, H. S. AlphaFold2 predicts the inward-facing conformation of the multidrug transporter LmrP. *Proteins: Structure, Function, and Bioinformatics* **89,** 1226–1228 (May 2021).

21. Debruycker, V. *et al.* An embedded lipid in the multidrug transporter LmrP suggests a mechanism for polyspecificity. *Nature Structural Molecular Biology* **27,** 829–835 (July 2020).

22. Steinegger, M. & Söding, J. MMseqs2 enables sensitive protein sequence searching for the analysis of massive data sets. *Nature Biotechnology* **35,** 1026–1028 (Oct. 2017).

23. Yan, R. *et al.* Mechanism of substrate transport and inhibition of the human LAT1-4F2hc amino acid transporter. *Cell Discovery* **7** (Mar. 2021).

24. Chin, D. & Means, A. R. Calmodulin: a prototypical calcium sensor. en. *Trends Cell Biol.* **10,** 322–328 (Aug. 2000).

25. Kuboniwa, H. *et al.* Solution structure of calcium-free calmodulin. en. *Nat. Struct. Mol. Biol.* **2,** 768–776 (Sept. 1995).

26. Houdusse, A., Love, M. L., Dominguez, R., Grabarek, Z. & Cohen, C. Structures of four Ca2+-bound troponin C at 2.0 Å resolution: further insights into the Ca2+-switch in the calmodulin superfamily. en. *Structure* **5,** 1695–1711 (Dec. 1997).

27. Zhang, M., Tanaka, T. & Ikura, M. Calcium-induced conformational transition revealed by the solution structure of apo calmodulin. en. *Nat. Struct. Mol. Biol.* **2,** 758–767 (Sept. 1995).

28. Kataoka, M., Persechini, A., Tokunaga, F. & Kretsinger, R. H. The linker of calmodulin lacking Glu84 is elongated in solution, although it is bent in the crystal. en. *Proteins* **25,** 335–341 (July 1996).

29. Igumenova, T. I., Lee, A. L. & Wand, A. J. Backbone and side chain dynamics of mutant calmodulin-peptide complexes. en. *Biochemistry* **44,** 12627–12639 (Sept. 2005).

30. Aykut, A. O., Atilgan, A. R. & Atilgan, C. Designing Molecular Dynamics Simulations to Shift Populations of the Conformational States of Calmodulin. *PLoS Computational Biology* **9** (ed Livesay, D. R.) e1003366 (Dec. 2013).

31. Pawson, T. & Schlessingert, J. SH2 and SH3 domains. *Current Biology* **3,** 434–442 (July 1993).

32. Musacchio, A., Gibson, T., Lehto, V.-P. & Saraste, M. SH3 — an abundant protein domain in search of a function. *FEBS Letters* **307,** 55–61 (July 1992).

33. Huse, M. & Kuriyan, J. The Conformational Plasticity of Protein Kinases. *Cell* **109,** 275–282 (May 2002).

34. Solheim, S. A. *et al.* Interactions between the Fyn SH3-domain and adaptor protein Cbp/PAG derived ligands, effects on kinase activity and affinity. *The FEBS Journal* **275,** 4863–4874 (Sept. 2008).

35. Matsumura, Y. *et al.* Transient Helical Structure during PI3K and Fyn SH3 Domain Folding. *The Journal of Physical Chemistry B* **117,** 4836–4843 (Apr. 2013).

36. Neudecker, P. *et al.* Identification of a Collapsed Intermediate with Non-native Long-range Interactions on the Folding Pathway of a Pair of Fyn SH3 Domain Mutants by NMR Relaxation Dispersion Spectroscopy. *Journal of Molecular Biology* **363,** 958–976 (Nov. 2006).

37. Chu, W.-T., Zhang, J.-L., Zheng, Q.-C., Chen, L. & Zhang, H.-X. Insights into the Folding and Unfolding Processes of Wild-Type and Mutated SH3 Domain by Molecular Dynamics and Replica Exchange Molecular Dynamics Simulations. *PLoS ONE* **8** (ed Ventura, S.) e64886 (May 2013).

38. Ollerenshaw, J. E., Kaya, H., Chan, H. S. & Kay, L. E. Sparsely populated folding intermediates of the Fyn SH3 domain: Matching native-centric essential dynamics and experiment. *Proceedings of the National Academy of Sciences* **101,** 14748–14753 (Oct. 2004).

39. Neudecker, P. *et al.* Structure of an Intermediate State in Protein Folding and Aggregation. *Science* **336,** 362–366 (Apr. 2012).

40. Yu, B. & Hunt, J. F. Enzymological and structural studies of the mechanism of promiscuous substrate recognition by the oxidative DNA repair enzyme AlkB. *Proceedings of the National Academy of Sciences* **106,** 14315–14320 (Aug. 2009).

41. Ergel, B. *et al.* Protein Dynamics Control the Progression and Efficiency of the Catalytic Reaction Cycle of the Escherichia coli DNA-Repair Enzyme AlkB. *Journal of Biological Chemistry* **289,** 29584–29601 (Oct. 2014).

42. Carvajal, R. D., Tse, A. & Schwartz, G. K. Aurora Kinases: New Targets for Cancer Therapy. *Clinical Cancer Research* **12,** 6869–6875 (Dec. 2006).

43. Pitsawong, W. *et al.* Dynamics of human protein kinase Aurora A linked to drug selectivity. *eLife* **7** (June 2018).

44. Bavetsias, V. & Linardopoulos, S. Aurora Kinase Inhibitors: Current Status and Outlook. *Frontiers in Oncology* **5** (Dec. 2015).

45. Sells, T. B. *et al.* MLN8054 and Alisertib (MLN8237): Discovery of Selective Oral Aurora A Inhibitors. *ACS Medicinal Chemistry Letters* **6,** 630–634 (May 2015).

46. Sloane, D. A. *et al.* Drug-Resistant Aurora A Mutants for Cellular Target Validation of the Small Molecule Kinase Inhibitors MLN8054 and MLN8237. *ACS Chemical Biology* **5,** 563–576 (May 2010).

47. Yang, Y. *et al.* Molecular dynamics and free energy studies on Aurora kinase A and its mutant bound with MLN8054: insight into molecular mechanism of subtype selectivity. *Molecular BioSystems* **8,** 3049 (2012).

48. Wilson, C. *et al.* Using ancient protein kinases to unravel a modern cancer drug's mechanism. *Science* **347,** 882–886 (Feb. 2015).

49. Occhipinti & Boron. Role of Carbonic Anhydrases and Inhibitors in Acid–Base Physiology: Insights from Mathematical Modeling. *International Journal of Molecular Sciences* **20,** 3841 (Aug. 2019).

50. Pilka, E. S., Kochan, G., Oppermann, U. & Yue, W. W. Crystal structure of the secretory isozyme of mammalian carbonic anhydrases CA VI: Implications for biological assembly and inhibitor development. *Biochemical and Biophysical Research Communications* **419,** 485–489 (Mar. 2012).

51. Dzubiella, J. Tightening and Untying the Knot in Human Carbonic Anhydrase III. *The Journal of Physical Chemistry Letters* **4,** 1829–1833 (May 2013).

52. Da Silva, G. M., Cui, J. Y., Dalgarno, D. C., Lisi, G. P. & Rubenstein, B. M. High-throughput prediction of protein conformational distributions with subsampled AlphaFold2. *gms_natcomms_1705932980_dat*[illegible] (2024).
